# A 2D ferroelectric vortex pattern in twisted BaTiO_3_ freestanding layers

**DOI:** 10.1038/s41586-023-06978-6

**Published:** 2024-02-14

**Authors:** G. Sánchez-Santolino, V. Rouco, S. Puebla, H. Aramberri, V. Zamora, M. Cabero, F. A. Cuellar, C. Munuera, F. Mompean, M. Garcia-Hernandez, A. Castellanos-Gomez, J. Íñiguez, C. Leon, J. Santamaria

**Affiliations:** 1 GFMC, Departamento Fisica de Materiales, Facultad de Fisica, Universidad Complutense, Madrid, Spain; 2 Laboratorio de Heteroestructuras con aplicación en spintrónica, Unidad Asociada UCM/CSIC, Madrid, Spain; 3 Instituto de Ciencia de Materiales de Madrid ICMM-CSIC, Madrid, Spain; 4 Materials Research and Technology Department, Luxembourg Institute of Science and Technology (LIST), Esch-sur-Alzette, Luxembourg; 5 ICTS Centro Nacional de Microscopia Electrónica ‘Luis Brú’, Universidad Complutense, Madrid, Spain; 6 Department of Physics and Materials Science, University of Luxembourg, Belvaux, Luxembourg

**Keywords:** Two-dimensional materials, Ferroelectrics and multiferroics, Surfaces, interfaces and thin films

## Abstract

The wealth of complex polar topologies^1–10^ recently found in nanoscale ferroelectrics results from a delicate balance between the intrinsic tendency of the materials to develop a homogeneous polarization and the electric and mechanical boundary conditions imposed on them. Ferroelectric–dielectric interfaces are model systems in which polarization curling originates from open circuit-like electric boundary conditions, to avoid the build-up of polarization charges through the formation of flux-closure^11–14^ domains that evolve into vortex-like structures at the nanoscale^15–17^ level. Although ferroelectricity is known to couple strongly with strain (both homogeneous^18^ and inhomogeneous^19,20^), the effect of mechanical constraints^21^ on thin-film nanoscale ferroelectrics has been comparatively less explored because of the relative paucity of strain patterns that can be implemented experimentally. Here we show that the stacking of freestanding ferroelectric perovskite layers with controlled twist angles provides an opportunity to tailor these topological nanostructures in a way determined by the lateral strain modulation associated with the twisting. Furthermore, we find that a peculiar pattern of polarization vortices and antivortices emerges from the flexoelectric coupling of polarization to strain gradients. This finding provides opportunities to create two-dimensional high-density vortex crystals that would enable us to explore previously unknown physical effects and functionalities.

## Abstract (web summary)

The stacking of freestanding ferroelectric perovskite layers with controlled twist angles results in a peculiar pattern of polarization vortices and antivortices that emerges from the flexoelectric coupling of polarization to strain gradients.

## Main

The persistence of ferroelectricity at the nanoscale level hinges on the compensation of the polarization-bound charges and depolarizing fields building up at the surfaces or interfaces. In ferroelectric films with metallic electrodes, the depolarizing fields can be screened by (free) charge accumulation and by the formation of domains^22^. The situation is even more pronounced in nanoscale ferroelectric samples with dielectric boundaries (including vacuum or insulating non-polar surface layers) in which the polarization can undergo a transition into vortex^15–17^ or more complex^1–9^ topological states, with rotational polar configurations persisting to small diameters in which polarization departs from the high-symmetry directions favoured by the lattice anisotropy^23^.

Mechanical boundary conditions^21^, as those imposed by interfacial strain, play an important part in determining the final polarization state, as they may combine with electric boundary conditions in non-trivial ways. Notably, the strong coupling of ferroelectricity with both homogeneous and inhomogeneous strain is at the origin of the effectiveness of mechanical boundary conditions in triggering unexpected effects, such as enhanced ferroelectricity in epitaxially strained layers^18^ or polarization switching under the strain gradients created by an atomic force microscopy tip pressing on the sample surface^24^. As it turns out, however, access to externally tunable strain patterns is in practice very limited.

In epitaxial thin films, mechanical boundary conditions are, to a large extent, immovably and solely determined by the atom-on-atom replication of the structure of the substrate by the growing film. Hence, although the interface with the substrate is subject to in-plane strains imposed by the lattice mismatch, the sample surface is in a zero-stress state, as there are no tractions acting on it. In epitaxial uniformly strained single-domain layers, internal elastic fields are homogeneous and rigidly imposed by these mixed boundary conditions. Inhomogeneous strain results typically from uncontrollable strain relaxation, misfit dislocations or ferroelastic domain formation^19^. The structural constraints imposed by epitaxy leave little or no room for the modification of the mechanical boundary conditions. Moreover, controllable shear or inhomogeneous strain patterns are commonly out of reach. This is the reason why, although on general grounds exotic ferroelectric states can be expected to result from the manipulation of mechanical boundary conditions, this scenario remains mostly unexplored.

In this paper, we demonstrate a strategy to engineer mechanical boundary conditions based on the strain modulation induced at the interface between two twisted freestanding oxide layers. In layered materials such as graphene or transition metal dichalcogenides, twisted bilayers have led to the emergence of unexpected collective states^25,26^. The weak van der Waals interlayer interaction in such twisted bilayers leads to inhomogeneous strain patterns with deformations up to about 1% (ref. ^27^). Extending the exploration to artificial twisted stacks of transition metal oxides with strong mixed ion–covalent bonds, however, has been hampered by the difficulty in isolating these systems in freestanding form. The recent reports on the fabrication of freestanding single crystalline oxide thin films^28–30^ showing exciting ferroelectric topologies^31,32^, and which can be handled in a way similar to van der Waals two-dimensional (2D) materials^33^, open up the possibility of stacking freestanding layers with arbitrary twist angles^34,35^ and thus design previously unknown strain patterns. Here we show that the lateral strain modulation caused by the interface matching between two twisted freestanding ferroelectric BaTiO_3_ (BTO) layers sets a mechanical boundary condition not attainable by epitaxial strain, and to a large extent controllable by the relative rotation angle. The nanoscale-modulated distribution of symmetric and antisymmetric shear strains yields a notable rotational polarization texture with alternating clockwise and anticlockwise vortices and antivortices, the distribution, spacing and size of which are controlled by the twist angle. First-principles simulations show that this complex configuration of highly localized symmetric and antisymmetric shears is concomitant with the ferroelectric vortex 2D modulation and constitutes a stable equilibrium state. The coupling between shear strain gradients and complex polarization texture is discussed in terms of a direct flexoelectric effect.

## Strain and polarization analysis of twisted bilayers

**Extended Data Fig. 1:**
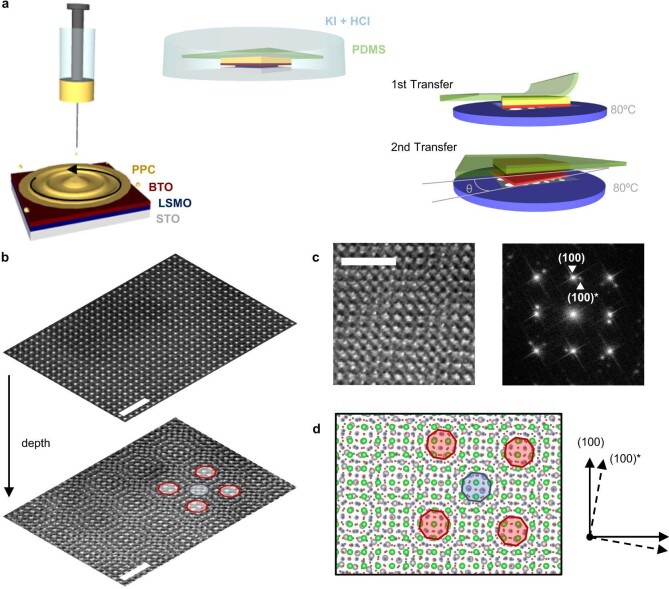
Fabrication of twisted freestanding BaTiO_3_ bilayers. a) Schematic of the deterministic two steps transfer process. b) Depth sectioning STEM-HAADF experiment of a twisted BaTiO_3_ bilayer stack focusing on the entrance surface of the stack (defocus = 0 nm) and the interface (defocus = −15 nm) c) Moiré structure formed at the interface between the BaTiO_3_ layers. A network of two distinct AA and AB motives is formed due to the twist. The image on the right shows the Fast Fourier Transform noting the reflections of both the top and bottom freestanding BaTiO_3_ films. The scale bar is 2 nm d) Model of the rigid atomic structure corresponding to two BaTiO_3_ lattices with a twist angle of 10.4° showing Ba atoms in green, Ti in blue and O in red for the top layer. Atoms in the bottom layer are in gray colors. AA and AB sites are marked in red and blue respectively.

BTO layers of thickness 15  nm epitaxially grown on (001) SrTiO_3_ substrates were delaminated to form twisted bilayer homojunctions with deterministic twist angles (see Methods and Extended Data Fig. 1a for a detailed description of the sample fabrication process). To study the structural properties of individual layers of the twisted bilayers, we performed a depth-sectioning high-angle annular dark-field (HAADF) scanning transmission electron microscopy (STEM) experiment (Methods) focusing on the top surface of the stack (defocus = 0 nm) and on the interface (defocus = −15 nm; see Extended Data Fig. 1b and Supplementary Movie 1). Twisted ferroelectric bilayers exhibit characteristic moiré features determined by the atomic coincidence pattern between the two layers (Extended Data Fig. 1c). We studied moiré structures formed in α = 3°, 6°, 10.4° and 50° twisted BTO bilayers.

**Fig. 1:**
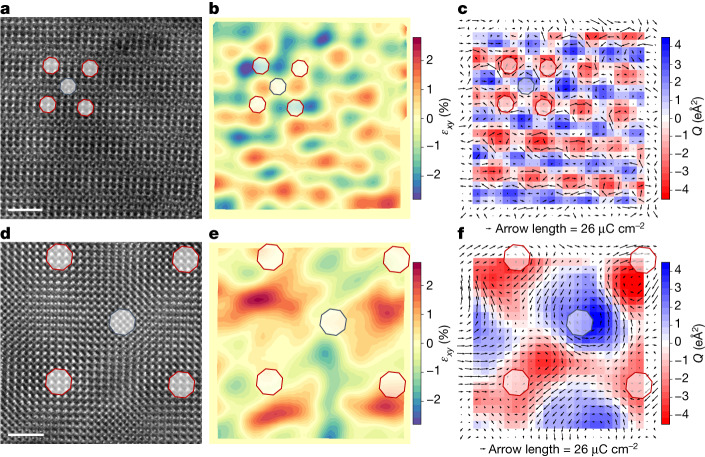
Strain and polarization modulations at twisted BTO bilayers. **a**, STEM-HAADF (planar view) image of a 10.4° twisted BTO bilayer stack focusing on the interface of the bilayer (defocus = −15 nm). **b**, Shear strain (*ε*_*xy*_ component of the lattice strain tensor) depicting a periodic strain modulation at the top BTO layer. **c**, Ti displacement map (*d*_Ti_ − ⟨*d*_Ti_⟩) after subtracting the homogeneous component (black arrows) measured on the top BTO layer corresponding to the same area superimposed on the toroidal moment (*Q*) of the ferroelectric polarization showing a network of clockwise (red) and anticlockwise (blue) vortices. Ti displacements (*d*_Ti_ − ⟨*d*_Ti_⟩) are amplified by a factor of 20 for clarity. **d**–**f**, The same analysis for a 3° twisted BTO bilayer. Red and blue octagons in all panels indicate sites with AA (AA sites) and AB (AB sites) stacking, respectively. The averaged polarization (modulus) is approximately 20 μC cm^−2^, close to the bulk BTO value. Scale bar, 2 nm (**a**,**b**).

Figure 1a,d shows the STEM-HAADF (planar view) images of 10.4° and 3° twisted bilayers. The moiré pattern shows two distinct plateau-like features at the highly (atom-on-atom) coincidental regions of both layers: AA (Ba on Ba and Ti on Ti) and AB (Ba on Ti and Ti on Ba) (see also Extended Data Fig. 1). The intralayer strain was measured on the top layer using the entrance surface focused image (defocus = 0; Methods). The emergence of a strongly spatially varying strain landscape with the same periodicity as the moiré lattice—and determined on the top layer—demonstrates the strong interaction between the two twisted layers. The resultant strain map shows a periodically modulated pattern of symmetric shear strains $({\varepsilon }_{xy}=\frac{1}{2}(\frac{\partial {u}_{x}}{\partial y}+\frac{\partial {u}_{y}}{\partial x}))$ with alternating positive and negative shear strain cores (see Fig. 1b,e for 10.4° and 3° twisted bilayers, respectively). In between the AA and AB sites of the moiré pattern, we find regions of maximum strain, named S sites hereafter, with nearly homogeneous positive and negative shears. The shear strain modulation shows the same periodicity as the moiré pattern, indicating that the strain results from a reconstruction in the top layer induced by the matching at the interface. An important remark is that such a periodic shear strain landscape is unique, as it cannot be attained, to the best of our knowledge, either by epitaxial strain or by any pattern of externally applied stresses.

To investigate how the strain modulation observed on the top layer of the twisted BTO bilayers affects the ferroelectric polarization, we measured the off-centring of the B-site cations in the individual unit cells (relative displacement of the B-site Ti cation from the centrosymmetric position, determined from the A-site Ba cations within the same unit cell). Twisted bilayers showed net in-plane polarization pointing in the in-plane [1, 1] direction, with a superimposed polar texture (Extended Data Fig. 4). Polar displacements in the range of 0.15–0.20 Å were obtained, which is consistent with what is found in bulk BTO. In BTO, the magnitude of the spontaneous polarization is known to be approximately constant regardless of the orientation it may present in the different ferroelectric phases this compound can adopt^36,37^, which suggests that the polarization at the surface must be largely confined to the plane. Yet, as planar views only probe the in-plane polarization, we resort to the polarization analysis of cross-section images to show that there is an out-of-plane polarization component (Methods and Extended Data Figs. 5 and 6).

The complex polar texture shown in the planar views of the twisted bilayers can be better assessed by subtracting the average polarization (**P** − ⟨**P**⟩) value in the image (Extended Data Fig. 4). The final polarization state results from the superposition of the nearly homogeneous polarization featured by isolated freestanding layers and the vortex-like modulation of the local ferroelectric potential imposed by the interface, yielding a peculiar 2D pattern of polarization waves^7^. The polarization (**P** − ⟨**P**⟩) maps in Fig. 1c,f show a continuous curling of the polar displacements, forming a periodic network of non-trivial topological structures with alternating vortices (AA and AB sites) and antivortices (S sites of the moiré pattern) of the local ferroelectric potential. We can describe the topological structure in terms of a non-zero toroidal moment^15–17^ parallel to the *z*-direction defined as ${\bf{Q}}=\frac{1}{2N}{\sum }_{1}^{N}{{\bf{r}}}_{i}\times {{\bf{P}}}_{i}$, where P_*i*_ is the local dipole moment located at r_*i*_ and *N* is the number of dipoles (cells).

The toroidal moment alternates sign periodically in diagonal directions of the moiré pattern (Fig. 1c,f) in a way determined by a periodic array of alternating clockwise and anticlockwise vortices in AA and AB sites, respectively. Ferroelectric vortices are topological objects characterized by a winding number *n* = +1 (Supplementary Note 1) regardless of their polarity (clockwise or anticlockwise). Values of the toroidal moment at the vortex sites depend on the size of the vortex and on the ferroelectric displacements (dipole moment). We obtain values similar to those reported for flat epitaxial BTO nanoparticles^17^. In the moiré pattern, vortices alternate with antivortices (sitting at S sites), which are topological structures with *n* = −1 winding number and zero toroidal moment.

Further confirmation of the presence of the vortex pattern is obtained from the strain and polarization analysis of the cross-section image, as it supplies complementary information to planar views (showing only in-plane polarization components) providing a lateral view of the vortex lattice, in which the in-plane (*P*_*x*_ along the [1, 0, 0] direction) and out-of-plane (*P*_*z*_ along the growth direction) components of the polarization vector can be probed, as well as the *ε*_*xx*_ in-plane component of the strain tensor. As discussed in Supplementary Note 2, an analysis of the spatial dependence of the polarization vector, after subtracting a local averaged polarization value (**P** − ⟨**P**⟩), showed a lateral modulation of both the *x* and *z* (growth direction) components fully consistent with the presence of the vortex lattice. Moreover, there is a close correspondence between the modulation periodicity of cross-section samples and planar views, suggesting that the vortex originating at the interface propagates in the layer in the direction of growth.

Reducing layer thickness to 8 nm stabilizes a vortex state with no homogeneous polarization. By contrast, as the thickness is increased, a homogeneous polarization component is observed as well as some degree of strain relaxation (Methods and Extended Data Fig. 8). Also, single layers show nearly homogeneous strain states with no indication of the polar topology observed in the twisted bilayers (Extended Data Fig. 3). The picture emerges that the inhomogeneous strain distribution imposed by the interface between the twisted ferroelectric layers results in vortex-like modulations of the local ferroelectric potential. Yet, for samples with sufficiently thin layers, a true vortex state with absent homogenous polarization is observed. Notably, there is a close correspondence between the vortex lattice and the distribution of shear strains underlying the moiré pattern. Clockwise or anticlockwise vortices are located at the AA and AB sites with nearly zero shear strain (albeit maximal rotational strain). By contrast, antivortices sit at the S sites with maximal shear strain (but nearly zero rotational strain).

## Theoretical description of the ferroelectric pattern

**Fig. 2:**
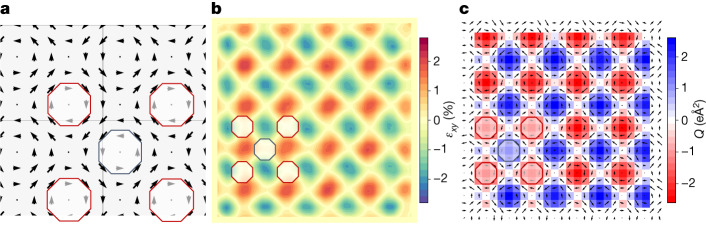
DFT model of a ferroelectric vortex lattice in BTO. **a**, Ti displacement map (black arrows) of the DFT-calculated model. DFT first-principles simulations did not show any homogeneous polarization component. **b**, Shear strain (*ε*_*xy*_ component of the lattice strain tensor) obtained from the DFT model. **c**, Ti displacement map (black arrows) superimposed on the toroidal moment (*Q*) of the ferroelectric polarization obtained from the DFT model. The arrows in **a** and **c** denote local dipoles as obtained from the product of local atomic displacements (with respect to a centrosymmetric reference structure) and Born effective charges. Ti displacements in **c** are amplified by a factor of 40 for clarity. Red and blue marks in all panels indicate the AA and AB stacking regions, respectively. Note that **a** is a magnification of the polarization map shown in **c**, to better see the topology of the polarization landscape.

To get further confirmation of this topological polar pattern, we resort to density functional theory (DFT), considering simplified (computationally tractable) simulated systems that are nevertheless relevant to our problem. More precisely, we work with a periodically repeated supercell composed of 6 × 6 × 1 elemental BTO units. As the starting point of our simulations, we use an atomic configuration that mimics as closely as possible the inhomogeneous polarization pattern observed experimentally in the 10° twisted layers. We then run a structural relaxation in which all atomic positions and supercell strains are allowed to evolve to minimize the DFT energy of the system. We obtain, as a stable solution, the polarization and strain maps shown in Fig. 2, in qualitative agreement with the experimental results of Fig. 1 and thus confirming the connection between the observed strain and dipole modulations. According to our simulations, this topological state is 9 meV per formula unit above the homogeneous orthorhombic phase with polarization along the [1, 1] diagonal. This relatively small difference is an upper bound (Methods) for the energy cost of deforming the trivial homogeneous state to acquire the topological features of Figs. 1 and 2. Hence, our calculations support the notion that interlayer interactions may suffice to induce the experimentally observed strain and dipole patterns.

## Strain–polarization coupling

Let us finally tackle this important question of what causes the peculiar inhomogeneous polarization textures in our layers. These complex quasi-periodic orders are controlled by the twist angle, which indicates that they are the result of interlayer interactions. Furthermore, it is apparent that the vortex- and antivortex-like dipole arrangements in Fig. 1c,f are correlated with the measured strain patterns of Fig. 1b,e. This suggests that, to understand these polar textures, it is reasonable to ignore the microscopic details of the couplings across the twisted interface and, instead, focus on how the observed elastic modulation affects the polarization. Ferroelectric perovskites such as BTO present strong electromechanical couplings that are potential candidates to explain our observations.

Let us begin by considering the simplest strain–polarization couplings. From well-established models of ferroelectric perovskites such as BTO (ref. ^38^), we know that a shear strain *ε*_*xy*_ > 0 typically favours a polarization oriented along the [1, 1] in-plane diagonal, whereas *ε*_*xy*_ < 0 leads to polarizations along [1, −1]; hence, we can expect *δP*_*x*_*δP*_*y*_ *∝* *ε*_*xy*_, where (*δP*_*x*_, *δP*_*y*_) refers to the inhomogeneous part of the measured polarization, as shown in Fig. 1c,f (and also in Fig. 2c). However, it is clear from our results that this relationship does not hold for the measured strains (Figs. 1b,e and 2b) and inhomogeneous polarizations (Figs. 1c,f and 2c), as we can, for example, find regions with *ε*_*xy*_ > 0 and an either positive or negative *δP*_*x*_*δP*_*y*_ product. A strong piezoelectric effect would also lead to *δP*_*x*_*δP*_*y*_ ∝ *ε*_*xy*_ and is not supported by our observations either. Hence, these are not the dominant couplings in our samples.

**Fig. 3:**
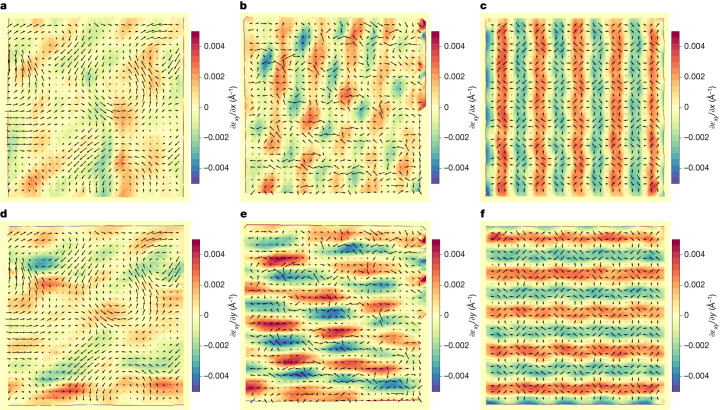
Shear strain gradients of twisted BTO bilayers. **a**–**c**, Derivative of the shear strain along the *x-*axis of a 3° twisted BTO bilayer (**a**) and a 10.4° twisted BTO bilayer (**b**) and a DFT-calculated model corresponding to 10° twisted layers (**c**). **d**–**f**, Derivative of the shear strain along the *y-*axis of a 3° twisted BTO bilayer (**d**) and a 10.4° twisted BTO bilayer (**e**) and a DFT-calculated model corresponding to 10° twisted layers (**f**). Ti displacement map (*d*_Ti_ − ⟨*d*_Ti_⟩) (black arrows) is superimposed on all images. Ti displacements are amplified for clarity by a factor of 20 in **a**, **b**, **d** and **e** and by a factor of 40 in **c** and **f**.

Next, we note that our measured strain maps feature large strain gradients with maximum values reaching ±4 × 10^7^ m^−1^ (Fig. 3). By direct flexoelectric coupling^39^, these gradients should yield a polarization change, the expected dominant effects being

1 $$\delta {P}_{x}\approx {\mu }_{xyxy}^{eff}\frac{\partial {{\epsilon }}_{xy}}{\partial y}$$

and

2 $$\delta {P}_{y}\approx {\mu }_{xyxy}^{eff}\frac{\partial {{\epsilon }}_{xy}}{\partial x},$$

where ${\mu }_{{xyxy}}^{\text{eff}}$ is an effective flexoelectric coefficient. Notably, from the measured strain gradients (Figs. 1b,e and 2b) and inhomogeneous polarization (Figs. 1c,f and 2c), we see direct support for a coupling of this kind in our results. We find that the regions with $\,\frac{\partial {{\epsilon }}_{xy}}{\partial x} > 0$, shown as red vertical fringes in Fig. 3, feature positive *δP*_*y*_ > 0; conversely, the regions with$\,\frac{\partial {{\epsilon }}_{xy}}{\partial x} < 0$, shown as blue vertical fringes in Fig. 3, show *δP*_*y*_ < 0. A similar relation holds for the $\frac{\partial {{\epsilon }}_{{xy}}}{\partial y}$ gradients and the *δP*_*x*_ component of the polarization.

**Fig. 4:**
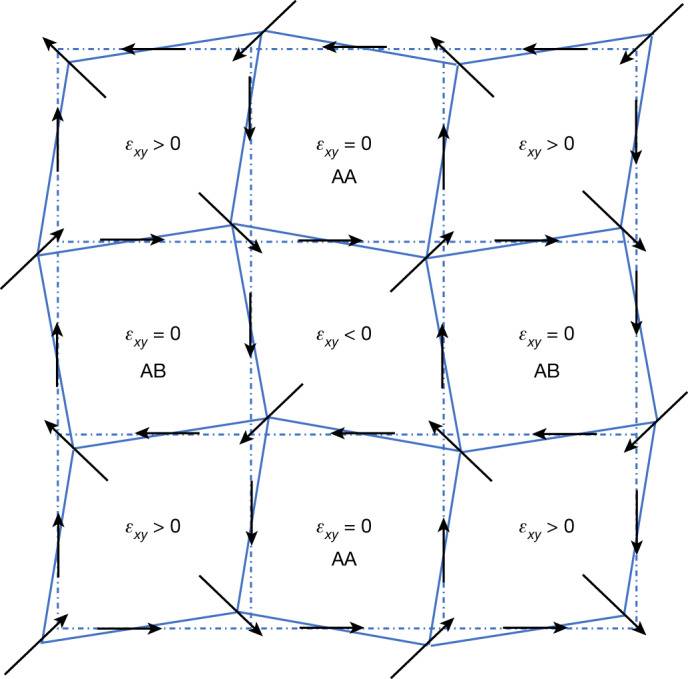
Pictorial view of the flexoelectric couplings. Sketch of the BTO layer, showing regions of approximately constant shear strain as cells of a periodic lattice. We indicate the analogues of the AA and AB sites discussed in the text. The black arrows stand for the polarization induced by the flexoelectric effect; these arrows are consistent with equations (1) and (2) for ${\mu }_{{xyxy}}^{\text{eff}} > 0$, and they present the vortices and antivortices observed experimentally. Note that the flexoelectric polarization can be intuitively understood from the symmetry breaking caused by the strain modulation. For example, at any given lattice point (shared by four cells, with four associated cell angles), we always find an arrow pointing towards the cell with the smallest (<90°) angle.

The relationship between strain and polarization patterns can be captured in a simple geometric manner. As shown in Fig. 4, the symmetry breaking caused by the shear (and rotational) strain modulation readily leads to the observed arrangement of polar vortices and antivortices. A local shear strain *ε*_*xy*_ ≠ 0 breaks the square symmetry of the cells of Fig. 4, yielding two large-angle corners and two small-angle corners. In this figure, the arrows (flexoelectric polarizations) are drawn assuming that the cations displace towards the small-angle corners, which naturally yields an antivortex-like dipole arrangement with zero curl of the polarization field centred at the cells with *ε*_*xy*_ ≠ 0. Correspondingly, polarization vortices (non-zero curl) form around the cells with *ε*_*xy*_ =  0.

Our experimental results enable us to estimate the effective flexoelectric coupling as

$${\mu }_{xyxy}^{eff}\approx \delta {P}_{x}{\left(\frac{\Delta {\varepsilon }_{xy}}{\Delta y}\right)}^{-1}\approx \frac{20\,\mu {\rm{C}}\,{{\rm{cm}}}^{-2}}{4\times {10}^{7}\,{{\rm{m}}}^{-1}}\approx 5\,{\rm{nC}}\,{{\rm{m}}}^{-1},$$

where the relatively small value obtained suggests that the large strain gradients in our samples are beyond the linear approximation (Methods).

It is also interesting to note that the second derivatives of the shear strain can be used to compute the expected curl of the polarization vector. From the flexoelectric coupling between strain gradients and polarization (equations (1) and (2)), the following relation holds:

$$\frac{\partial {P}_{x}}{\partial y}-\frac{\partial {P}_{y}}{\partial x}={\mu }_{{xyxy}}^{{\rm{eff}}}\left(\frac{{\partial }^{2}{{\epsilon }}_{{xy}}}{\partial {y}^{2}}-\frac{{\partial }^{2}{{\epsilon }}_{{xy}}}{\partial {x}^{2}}\right),$$

closely captured by experimental results (see Extended Data Fig. 9 showing the second derivatives of the strain gradient and Extended Data Fig. 10 showing the curl of the polarization).

Finally, it is worth noting the recent claims of moiré-induced ferroelectricity in twisted heterobilayers of van der Waals materials^40,41^. Moreover, polar domains have been observed without an overall ferroelectric response^42^ as well as switching of polarization in a stacking domain of hexagonal boron nitride using van der Waals sliding^43^. Theoretical studies^44^ conclude that a switchable polarization that is remanent in zero applied electric field remains to be demonstrated to substantiate the claim of ferroelectricity. By contrast, in our case, the freestanding layers possess a robust ferroelectric ground state and the polar topology results from its modulation by the moiré interface strain pattern. Moreover, inducing strain and rotational polarization landscapes in nanometre-thick ferroelectric layers avoids artefacts appearing in twisted ultrathin (atomically thick) graphene layers, in which open questions remain on the strain induced by the moiré superlattice and its relaxation^27^.

## Summary and outlook

In summary, we have found that it is possible to induce non-trivial ferroelectric textures in twisted freestanding ferroelectric layers. The driving force is the couplings across the interface between the twisted layers—that is, the mechanical boundary conditions they effectively impose on each other. These couplings cause large strain gradients in the ferroelectric layers, which in turn yield vortex-like modulations of the homogeneous polarization state by flexoelectric effect. Accordingly, we find that the periodicity of the 2D vortex pattern can be largely tuned by controlling the twisting angle.

This provides opportunities enabled by the unique modulations that are possible in moiré bilayers to explore physical effects and functionalities, as well as whether they could host topologies such as the hopfions and Solomon rings recently found in BiFeO_3_ nanocrystals^45^. The highly correlated topological pattern with vortices and antivortices is reminiscent of the square lattice of merons, objects with *n* = ½ topological number existing only in lattices, observed in chiral magnets with magnetic anisotropy^32,46^. At variance with previous ferroelectric textures found in ferroelectric films confined in the growth direction, our polar landscape is 2D and highly tunable by controlling the twisting angle of the bilayer and, thus, it is more amenable for applications in high-density ferroelectric memories reaching the Gbit in^−2^ limit enabled by small few nm^2^ topological objects^31^. In a more fundamental direction, we believe that proximity interactions between layers with different ferroic orders may strongly depend on the twist angle. Twisted heterolayers will certainly provide opportunities for exploring previously unknown phenomena in surface physics and chemistry.

## Methods

### Fabrication of freestanding perovskite films

BTO layers of thickness 15  nm were grown onto La_0.7_Sr_0.3_MnO_3_ (LSMO)-buffered (100) SrTiO_3_ substrates using pure oxygen sputtering technique at high pressures (3.2 mbar) (ref. ^47^). This technique produces highly epitaxial growth with sharp interfaces and negligible stoichiometry deviations (see the next section on EELS analysis). The LSMO acts as a sacrificial layer that allows the release of the BTO layer on immersion in a selective KI + HCl etchant (33 mg of KI in an HCl solution 1.8% volume concentration) for an average time of 3 days (ref. ^48^). Before immersion, a polypropylene carbonate (PPC; Sigma Aldrich) solution (10 g of PPC per 100 ml of anisole) was spin‐coated (7,500 rpm for 30 s) onto the pristine BTO sample. After curing the PPC at 70 °C for 10 min in a hot plate, the sample was adhered to a commercial polydimethylsiloxane (PDMS; Gel‐Film WF 4 × 6.0 mil by Gel‐Pak) stamp. The sample was dipped into the etchant solution for 3 days, a period within which the substrate typically detaches on its own from the film supported by the polymer. The sample was then washed in deionized water for 30 s. The supporting polymer enables the manipulation of the freestanding oxide layer until transferred onto holey Si_3_N_4_ membrane grids for STEM observation. The transfer was performed by keeping the grid onto a Peltier plate at 80 °C to favour the transfer of the entire (about 5 × 5 mm^2^ size) BTO layer. After removing the PDMS, the membranes were dipped in acetone and isopropyl alcohol to remove the remaining PPC and clean the surface of the freestanding oxide layer. The deterministic assembly of the twisted bilayer was performed by mounting the first layer on a rotatable platform under the microscope in a custom-made layer-transfer setup. Layer edges (parallel to substrate edges along the [100] directions) were aligned with reference markers of the sample holder. The second layer can be placed on the top rotated with respect to the first using layer edges (which are parallel to [100] BTO crystallographic directions) to determine the angle. The twist angle was set with an accuracy of 1°. A more precise value of the twist angle (accuracy of 0.1°) can be measured from the shift between the fast Fourier transform (FFT) spots of electron microscopy images. Supplementary Fig. 1 shows an optical microscopy image of a twisted bilayer transferred onto a gold-capped silicon wafer. Note the large area (around mm^2^ size) of the sample. Although some wrinkles can be observed, they are separated by sufficiently large distances to have extensive areas in which the two layers superpose flat on top of the other.

Atomic force microscopy observation on the surfaces of twisted bilayers showed similar small roughness, taking values in the range of 0.5 nm (rms) over micron-sized distances (Supplementary Fig. 2).

### Scanning transmission electron microscopy

STEM characterization was carried out using a JEOL JEM-ARM 200cF aberration-corrected electron microscope equipped with a cold field emission gun and a Gatan Quantum spectrometer, operated at 200 kV. Depth-sectioning STEM-HAADF was performed by acquiring atomic-resolution STEM-HAADF images as a function of defocus^49,50^, enabling us to probe different depths of the sample and discriminate between the top and bottom layers of the stack. STEM-HAADF images were acquired using a 30-mrad probe forming aperture semiangle and a HAADF detector collection semiangle of 70–200 mrad.

### Quantitative EELS analysis

For EELS characterization, an electron energy-loss spectrum was acquired for every pixel while scanning the beam with an acquisition time of 1 s per pixel. For acquisition, we used Dual-EELS mode with a 0.25-eV dispersion that enabled us to simultaneously record the zero-loss-peak and the BTO core-loss edges. For EELS elemental composition analysis, we performed a model-based quantification, including plural scattering^51,52^, using the routines available in the Gatan Digital Micrograph suite. Supplementary Fig. 3 shows the relative Ti–O concentration profiles. Our analysis shows that the as-grown samples have stoichiometric Ti and O concentrations.

### Analysis of strain and polarization in twisted bilayers

Nanometre-thick (8–30 nm) BTO layers delaminated to form twisted bilayer homojunctions with deterministic twist angles were transferred onto holey Si_3_N_4_ membranes for electron microscopy imaging. To study the structural properties of individual layers of the twisted bilayers, we performed a STEM-HAADF experiment (see the previous section). Focusing on the entrance surface of the stack (defocus = 0 nm), we observed the typical structure of a BTO perovskite that corresponds to the top layer. The moiré contrast was shown by changing the defocus to reach the interface of the twisted bilayer (defocus = −15 nm) (Extended Data Fig. 1b). A further increasing defocus brings the bottom layer in focus, which appears rotated by the twist angle of the bilayer. Twisted ferroelectric bilayers exhibit characteristic moiré features determined by the atomic coincidence pattern between the two layers (Extended Data Fig. 1c). The 10.4° twist angle, determined from the FFT image, is homogeneous along the fabricated sample and close to the nominal 10° rotation of the films during the deterministic transfer process. The FFT shows the spots from both the top and bottom twisted BTO layers; for clarity, we denote the directions corresponding to the twisted layer forming the moiré pattern as (100)* and (010)*. The moiré pattern shows two distinct (plateau-like) features at the highly (atom-on-atom) coincidental regions of both layers, marked as AA and AB in Extended Data Fig. 1b and on the rigid atomic model shown in Extended Data Fig. 1d. Around the AA sites, there is AA stacking (Ba on Ba, Ti on Ti and O on O) between the top and bottom layers, whereas AB sites show an AB stacking (Ba on Ti and Ti on Ba) for the Ba and Ti cations of the twisted layers while preserving the AA stacking for the O anions.

We analysed strain using the Peak Pairs Analysis (PPA) software package (HREM Research) for Digital Micrograph^53^. We analysed the STEM-HAADF images of 3° and 10.4° twisted BTO bilayer stacks acquired focusing on the entrance surface of the stack (defocus = 0 nm). To improve the precision of the analysis, the scanning direction was rotated off the crystallographic axes of BTO. For the analysis, we performed a Bragg filter selecting the two main reflections along the (100) and (010) directions as the base vectors. The peak positions were then determined on the filtered image, and the relative displacement fields (*u*_*x*_, *u*_*y*_) of the measured lattice with respect to a reference lattice area were calculated. In this case, we used the whole image as a reference area. Finally, the components of the strain tensor were calculated from the displacement fields as ${\varepsilon }_{{xx}}=\frac{\partial {u}_{x}}{\partial x}$, ${\varepsilon }_{{yy}}=\frac{\partial {u}_{y}}{\partial y}$, ${\varepsilon }_{{xy}}=\frac{1}{2}\left(\frac{\partial {u}_{x}}{\partial y}+\frac{\partial {u}_{y}}{\partial x}\right)$ and ${\omega }_{xy}=\frac{1}{2}\left(\frac{\partial {u}_{y}}{\partial x}-\frac{\partial {u}_{x}}{\partial y}\right)$.

**Extended Data Fig. 2:**
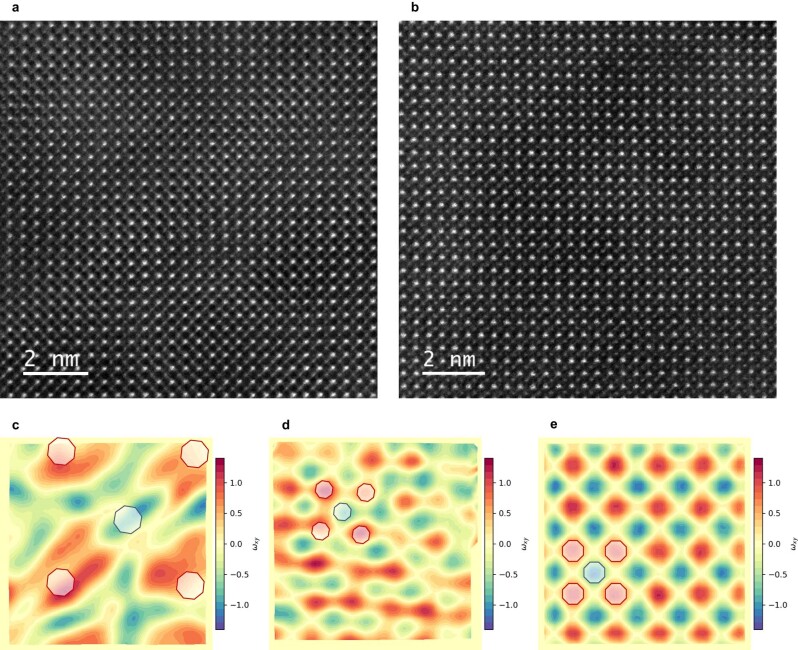
(a), (b) HAADF-STEM raw (unprocessed) planar view images of twisted BaTiO_3_ bilayers. a) STEM-HAADF image of a 3° twisted BaTiO_3_ bilayer stack focusing on the surface of the bilayer (defocus = 0 nm). b) STEM-HAADF image of a 10.4° twisted BaTiO_3_ bilayer stack focusing on the surface of the bilayer (defocus = 0 nm). **c)-e)** Antisymmetric component of the strain tensor (ω_xy_) measured on a) experimental HAADF image of a 3° twisted BaTiO_3_ bilayer b) experimental HAADF image of a 10.4° twisted BaTiO_3_ bilayer and c) model image obtained using a DFT model of a 10° twisted BaTiO_3_ bilayer. Red and blue octagons indicate sites with AA (AA-sites) and AB (AB-sites) stacking respectively.

**Extended Data Fig. 3:**
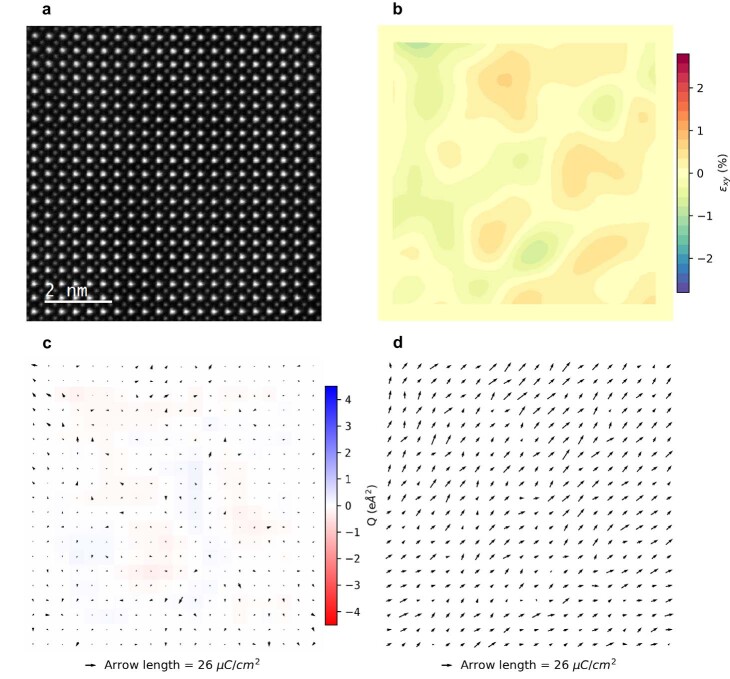
Strain and polarization analysis of a single BaTiO_3_ freestanding film. a) STEM-HAADF image (planar view) of a 15 nm BaTiO_3_ freestanding film along the [001] direction. b) Shear strain (*ε*_*xy*_ component of the lattice strain tensor) c) Ti displacement ($${d}_{{Ti}}-\left\langle {d}_{{Ti}}\right\rangle )$$ map (black arrows) corresponding to the same area superimposed to the toroidal moment (Q) of the ferroelectric polarization. d) Ti displacement ($${d}_{{Ti}})$$ showing the homogeneous polarization of a single BaTiO_3_ layer. Ti displacements in (c) and (d) are amplified by a factor of 20 for clarity.

Extended Data Fig. 2a,b shows (raw) unprocessed images of the top layer of both bilayers. Intralayer strain was measured on the top layer (defocus = 0). Apart from the symmetric shear strains (*ε*_*xy*_), strain analysis included the antisymmetric components of the strain tensor (*ω*_*xy*_) associated with local rotations of the perovskite lattice (Extended Data Fig. 2c,d). Control experiments on single BTO freestanding layers show a nearly homogeneous strain distribution (Extended Data Fig. 3), making it clear that the complex strain maps obtained in the bilayers originate at the stacking of the twisted layers. Note that there is a maximal atom-on-atom coincidence between the twisted layers at the AA and AB sites; we find very small shear strains in those areas.

To determine the ferroelectric polarization, the atomic positions of both A-site Ba and B-site Ti cations were measured on fast-Fourier-filtered STEM-HAADF images of twisted BTO bilayer stacks acquired focusing on the entrance surface of the stack (defocus = 0 nm). To precisely determine the atomic positions, we performed a 2D Gaussian fitting using Atomap^54^. Polarization was calculated from the off-centring of the B-site Ti cations in the individual unit cells (relative displacement of the B-site Ti cation from the centrosymmetric position, determined with the A-site Ba cations within the same unit cell)^55^.

**Extended Data Fig. 4:**
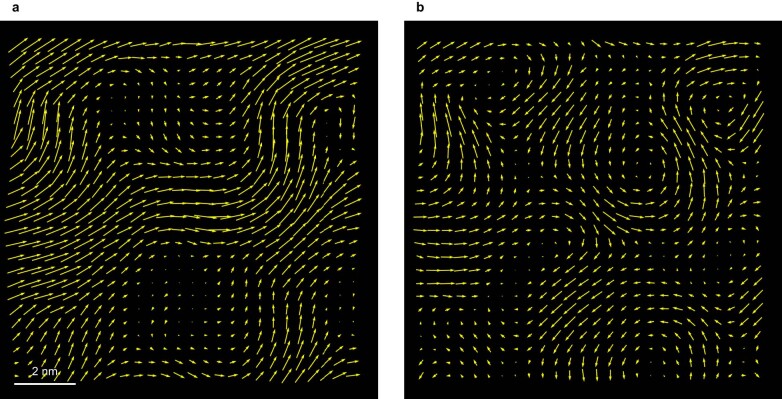
Polarization analysis of a 3° twisted BaTiO_3_ bilayer stack focusing on the top surface (defocus = 0 nm). a) Ti displacement map measured on the image in Fig. 1f showing the polarization before subtracting the mean polarization value of the analyzed region. b) Ti displacement map after subtracting the mean polarization value. Ti displacements are amplified by a factor of 20 for clarity.

Extended Data Fig. 4 shows the polarization analysis of a 3° twisted BTO bilayer. Arrows on the left panel (whose length scales with the BTO bulk ferroelectric moments) feature a pattern of polarization waves. The array of ferroelectric vortices can be better observed after subtracting the average polarization (**P** − ⟨**P**⟩) value in the image (Extended Data Fig. 4, right).

The effect of image distortions due to aberrations and artefacts due to FFT analysis and electron channelling effects^56^ through the whole bilayer system is described in Supplementary Note 3.

### Cross-section samples for the analysis of out-of-plane polarization

**Extended Data Fig. 5:**
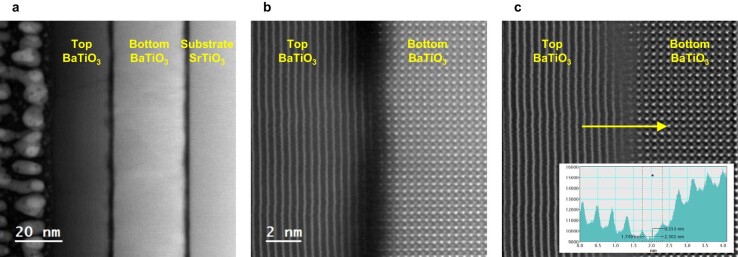
Cross-sectional HAADF-STEM images of a 10° twisted BaTiO_3_ bilayer. a) STEM-HAADF (cross-section) image of a 10° twisted BaTiO_3_ bilayer transferred over a SrTiO_3_ substrate. The bottom layer of the bilayer was aligned with the substrate. b) STEM-HAADF image of the interface between the top and bottom layers of the stack. c) Fourier-filtered image from (b). The inset shows an intensity profile along the yellow arrow depicting a distance of the order of one unit cell between the top and bottom layers of the stack.

The origin of the inhomogeneous strain is the interfacial proximity-induced physical couplings between both ferroelectric layers enabled by a narrow interface between the twisted layers. Extended Data Fig. 5 shows cross-sectional STEM-HAADF images of a 10° twisted BTO bilayer. The width of the interface measured from intensity profiles across the interface in mask-filtered inversed FFT images (Extended Data Fig. 5c) is of the order of a single unit cell. Note that the dark contrast at the interface of the stack is similar in recently reported images of twisted SrTiO_3_ membranes^35^ and can be explained by a reduced channelling effect at the interface^57^. As demonstrated by cross-section high-resolution microscopy images, there is a quite ‘intimate’ contact between the layers that enables the interaction between the layers^58^, which is expected to be strong for our polar oxides^59^ and may survive even across the adatom layers^60^. Occasionally, we find regions with increased interface width resulting from disorder or adsorbates, in which neither the moiré pattern nor the strain modulation is observed.

**Extended Data Fig. 6:**
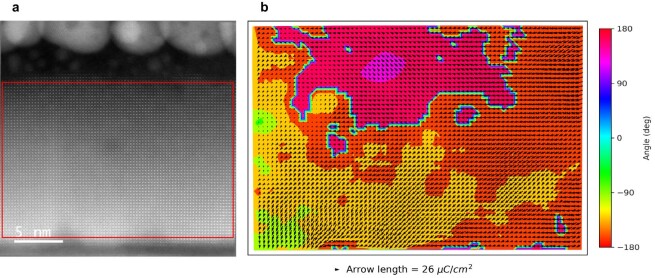
Polarization analysis of a cross-section image of a 10° twisted BaTiO_3_ bilayer transferred onto a SrTiO_3_ substrate. a) STEM-HAADF image along the [0,1,0] direction of the top layer in a 10° twisted BaTiO_3_ bilayer. b) Ti displacement ${(d}_{{Ti}})$ map (black arrows) measured on the marked region in (a) at the top BaTiO_3_ layer. The color map shows the angle of the polarization measured respect to the positive direction (left to right) of the horizontal axis. The cross-sectional sample was capped with an evaporated gold layer (top part of the image) to protect the twisted freestanding layers during sample preparation.

Extended Data Fig. 6 shows a polarization map of the top layer of the bilayer of Extended Data Fig. 5. Note that although at the top surface there are large regions with in-plane polarization, at the interface (bottom part of the image) polarization is mostly diagonal. The finding of an out-of-plane polarization close to the interface suggests the nucleation of a rhombohedral-like phase with polar displacements approximately in the ⟨1, 1, 1⟩ direction. (Strictly speaking, this phase is probably monoclinic.) This is not completely unexpected given the presence of the bottom interface in which the defects and/or adsorbates may favour the occurrence of an out-of-plane polarization component.

**Extended Data Fig. 7:**
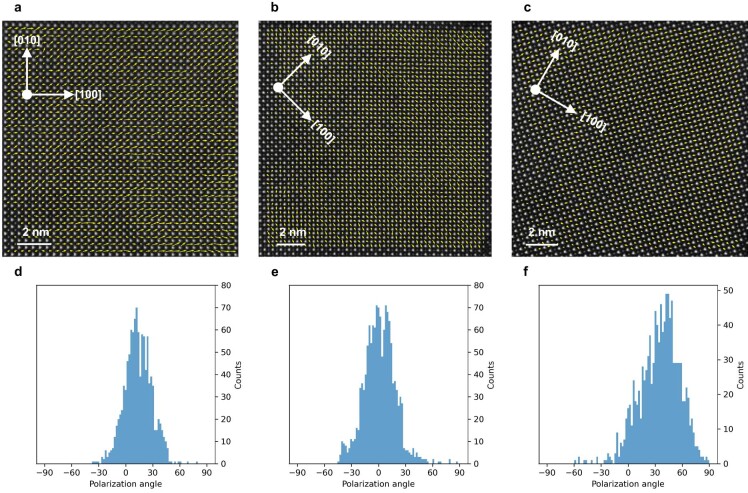
Polarization analysis of different regions of a single BaTiO_3_ freestanding film. a), b), c) STEM-HAADF images of different regions of a 15 nm BaTiO_3_ freestanding film acquired with different scanning directions. Each image was taken with scan directions to avoid possible artefacts. Ti displacement maps (yellow arrows) are superimposed to the images and displacements are amplified by a factor of 20 for clarity. Notice that in panel a) and b) in-plane polarization is mainly in the [1,0] direction, while in panel c) it points in [1,1] direction. d), e), f) Histograms of the polarization direction corresponding to the images in a), b), c). Histogram directions are set so the [100] direction corresponds to a 0° polarization angle in all cases. The offset found in region (a) is not related to the scan direction but it results from the presence of a small domain pointing along the [11] direction on the left part of the image.

By contrast, the net in-plane polarization at the surface of the top layer indicates that our layers present the tetragonal and orthorhombic ferroelectric phases that also occur in the bulk material. BTO crystals present a tetragonal structure at room temperature, which would suggest a polarization along the [1, 0] or [0, 1] direction also found in our layers. Single BTO freestanding layers showed averaged in-plane polarization in the [1, 0] direction, although occasionally [1, 1] orthorhombic domains were also observed (see Extended Data Fig. 7, which shows the histogram plots of the ferroelectric polarization direction in single BTO freestanding layers). Given the proximity to the bulk tetragonal to orthorhombic transition (which occurs at 278 K, implying that these phases have very similar free energies at ambient conditions) and the fact that the existing interfaces may cause more out-of-plane relaxations, the observed ferroelectric domains suggesting nucleation of different phases seem perfectly acceptable, as they may be stabilized by any of many factors distinguishing our BTO layers from the bulk compound. It is interesting to remark that single layers, apart from the large domains with homogeneous ferroelectric polarization discussed above, also show nearly homogeneous strain states with no indication of the polar topology observed in the twisted bilayers (see Extended Data Fig. 3, which shows a similar analysis for a single layer to that shown in Fig. 1 for the twisted bilayers).

### Layer thickness dependence

**Extended Data Fig. 8:**
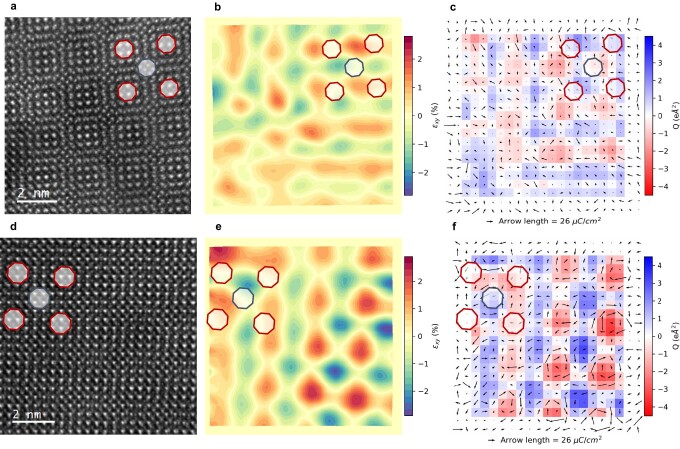
Strain and polarization modulations of 11° twisted BaTiO_3_ bilayers of different thickness. a) STEM-HAADF (planar view) image of a 11° twisted BaTiO_3_ bilayer stack with 30 nm thick layers focusing on the interface of the bilayer (defocus = −30 nm). b) Shear strain (*ε*_*xy*_ component of the lattice strain tensor) depicting a periodic strain modulation at the top BaTiO_3_ layer. c) Color map of the toroidal moment (Q) of the ferroelectric polarization corresponding to the same area showing a network of clockwise (red) and anticlockwise (blue) vortices. Superposed is the Ti displacement (${d}_{{Ti}}-\left\langle {d}_{{Ti}}\right\rangle )$ map (black arrows) measured on the top BaTiO_3_ layer after subtracting the homogeneous component. Ti displacements are amplified by a factor of 20 for clarity. d), e), f) show the same analysis for a 11° twisted BaTiO_3_ bilayer with 8 nm thick individual layers (defocus = −8 nm). Notice that the arrow map in f) has been constructed without subtracting the homogeneous polarization. Red and blue marks in all panels indicate the AA and AB stacking sites, respectively.

**Extended Data Fig. 9:**
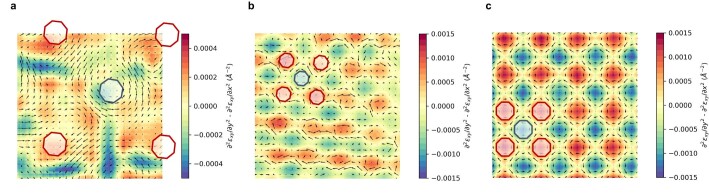
Second derivatives of the shear strain. **:** $\frac{{{\boldsymbol{\partial }}}^{{\bf{2}}}{{\boldsymbol{\epsilon }}}_{{\boldsymbol{xy}}}}{{\boldsymbol{\partial }}{{\boldsymbol{y}}}^{{\bf{2}}}}-\frac{{{\boldsymbol{\partial }}}^{{\bf{2}}}{{\boldsymbol{\epsilon }}}_{{\boldsymbol{xy}}}}{{\boldsymbol{\partial }}{{\boldsymbol{x}}}^{{\bf{2}}}}$ (proportional to curl of the polarization vector) for a) a 3° twisted BaTiO_3_ bilayer b) for a 10.4° twisted BaTiO_3_ bilayer and c) for a model image obtained from DFT calculation of a 10° twisted BaTiO_3_ bilayer. Notice the similarity with the toroidal moment shown in Fig. 1 showing a network of clockwise (red) and counterclockwise (blue) vortices at AA and AB sites respectively (marked with octagons). Ti displacement map (black arrows) are superimposed to all images. Ti displacements are amplified by a factor of 20 in (a, b) and 40 in (c).

**Extended Data Fig. 10:**
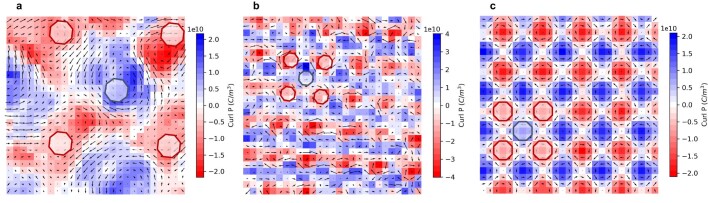
Curl of the polarization vector. for a) experimental HAADF image of a 3° twisted BaTiO_3_ bilayer b) experimental HAADF image of a 10.4° twisted BaTiO_3_ bilayer and c) model image obtained using a DFT model of a 10° twisted BaTiO_3_ bilayer. Notice the similarity with the toroidal moment shown in Fig. 1 showing a network of clockwise (red) and counterclockwise (blue) vortices at AA and AB sites respectively (marked with octagons). Ti displacement map (${d}_{{Ti}}-\left\langle {d}_{{Ti}}\right\rangle )$ after subtracting a homogeneous component (black arrows). Ti displacements are amplified for clarity by a factor of 20 in (a, b) and 40 in (c).

The thickness dependence of the observed strain–polarization pattern provides an important clue on the origin of the effect (Extended Data Fig. 8, which shows the polar topology of bilayers with 30-nm and 8-nm thick BTO layers with 11° twist angle). Owing to the focus depth of our microscope, 8 nm thickness is the lower limit allowing to resolve the atomic positions of the two layers independently (Supplementary Fig. 4). The larger thickness of the individual layer results in smaller values of the shear strain at the antivortex sites (around 1% for the 30-nm sample instead of about 2.8% for the bilayer with 15-nm layers) and also smaller values of the toroidal moment at vortex sites after subtracting the homogeneous polarization (approximately 1.5 eÅ^2^ for the 30-nm sample instead of 4 eÅ^2^ found for the 15-nm bilayer). On the contrary, in the 8-nm sample, the polar topology is observed even without subtracting the homogeneous polarization, which produces negligible changes in the polarization landscape. Also, larger average values of shear strain (2.9%) and toroidal moment (3.5 eÅ^2^), comparable to the 15-nm sample, are obtained in the thinner sample. This demonstrates that the pattern of shear strains induced by the relative rotation of the layers stabilizes a vortex state for sufficiently thin (8 nm) layers. As the thickness increases, a homogeneous polarization component is observed.

### First-principles calculations

We performed DFT calculations as implemented in the Vienna Ab initio Simulation Package (VASP)^61,62^. We used the Perdew–Burke–Ernzerhof formulation for solids (PBEsol)^63^ implementation of the generalized gradient approximation for the exchange-correlation functional. The atomic cores are treated within the projector-augmented wave approach^64^, considering the following states explicitly: 5*s*, 5*p* and 6*s* for Ba; 3*p*, 4*s* and 3*d* for Ti; and 2*s* and 2*p* for O. We used a 500-eV energy cutoff for the plane-wave basis set. The simulation cells consisted of 6 × 6 × 1 perovskite unit cells and were computed using a 1 × 1 × 4 Monkhorst–Pack^65^ k-point grid. The structures were fully relaxed until the residual forces fell below 0.01 eV Å^−1^ and the residual stresses fell below 0.001 GPa.

Let us stress that our DFT simulations correspond to the limit of very low temperature (formally, 0 K). Thus, the computed energy differences—that is, the 9 meV per formula unit separating the monodomain ferroelectric state from the vortex–antivortex structure—can be taken as an upper bound for the relevant free energy difference at room temperature. (In essence, the calculated energy difference comes from the ferroelectric domain walls—the energy of which is known to decrease on heating—and the inhomogeneous strain modulation—which is imposed by the interlayer couplings.) Note also that in our simulations we treat the monodomain and vortex–antivortex configurations as two separate cases, whereas in experiment (except in the thinnest layers) the topological features are a relatively small modulation of the homogeneous state. For this reason too, the computed energy difference is an upper bound for the actual energy cost of inducing (relatively small) topological features superimposed on the homogeneous state. Hence, our DFT results indicate that the experimentally observed topological structure can be easily accessed and physically sound.

The above considerations also explain the quantitative differences between the experimentally observed local polarizations (of about 0.2 C m^−2^) and those obtained in the simulated vortex–antivortex state (of about 0.1 C m^−2^). Although the former is associated with a largely homogeneous polar state, the latter corresponds to a configuration in which the total polarization changes sign in length scales of a few unit cells. It is natural to find local polarizations of reduced magnitude in such a strongly inhomogeneous state, as this yields smaller polarization gradients and a decrease in the associated energy penalty.

Finally, let us remark that simulating directly the perturbed homogeneous state would require DFT relaxations constrained to respect the experimentally observed inhomogeneous strain pattern; these calculations would involve several non-trivial assumptions and technical complications, and we did not pursue them here.

### Quantitative estimates of the flexoelectric coefficient

Our quantitative measurements enable us to compute strain gradients and polarization modulations and, thus, estimate the effective linear flexoelectric coefficient ${\mu }_{{xyxy}}^{\text{eff}}$. We have

$${\mu }_{xyxy}^{{\rm{eff}}}\approx \delta {P}_{x}{\left(\frac{\Delta {{\epsilon }}_{xy}}{\Delta y}\right)}^{-1}\approx \frac{20\,\mu {\rm{C}}\,{{\rm{cm}}}^{-2}}{4\times {10}^{7}\,{{\rm{m}}}^{-1}}\approx 5\,{\rm{nC}}\,{{\rm{m}}}^{-1},$$

which is substantially smaller than the typical experimental results for bulk BTO at room temperature (values between 0.15 μC m^−1^ and 3.3 μC m^−1^ have been reported^66–68^). However, we believe that this disagreement is probably not that surprising, for two main reasons: (1) our twisted layers are very different from the model bulk materials described in the literature; (2) the strain gradients we measure are orders of magnitude larger than the ones used for measurements of flexoelectric coefficients. Concerning the first point, our constrained BTO layers might be electrically stiffer than the bulk material and thus present a smaller flexoelectric response. (The magnitude of the flexoelectric coupling is known to be proportional to the magnitude of the dielectric response^21^.) Concerning the second point, note that to determine flexoelectric coefficients experimentally, the considered strain gradients are chosen intentionally small, of the order of 1 m^−1^ (ref. ^69^). Strain gradients of the order of 8 × 10 m^−1^—as those associated with ferroelastic domains^19^—are considered to be very large. The gradients in our samples are even larger, by almost two orders of magnitude. This suggests that the flexoelectric effects at play in our samples must be strongly non-linear. Hence, it is not justified to compare our estimated effective linear coupling with the strictly linear effects reported in the experimental literature.

Moreover, we may have differences because of surface contributions to the flexoelectric effect^70^. Furthermore, let us note that there are theoretical predictions yielding *μ*_*xyxy*_ values around 0.08 nC m^−1^ for BTO (ref. ^71^)—that is, a smaller effect than what we estimate. Shedding further light on these issues would be a great challenge, for both experiment and theory, and is beyond the scope of the present work.

## Online content

Any methods, additional references, Nature Portfolio reporting summaries, source data, extended data, supplementary information, acknowledgements, peer review information; details of author contributions and competing interests; and statements of data and code availability are available at 10.1038/s41586-023-06978-6.

### Supplementary information

Supplementary Information Supplementary Notes 1–3 and Supplementary Figs 1–11.

Supplementary Video 1 Depth-sectioning experiment. For the depth-sectioning experiment, a series of 20 STEM-HAADF images of a 3° twisted 15-nm BTO bilayer was acquired by continuously changing the defocus by a step of 2 nm from the entrance surface of the stack to the exit surface of the stack.

## Data Availability

The raw data shown in the main figures are available at Zenodo (10.5281/zenodo.10439374). Other data that support the findings of this study are available from the corresponding authors upon reasonable request.
