# Supplementary material for: A 2D ferroelectric vortex pattern in twisted BaTiO3 freestanding layers
Source: Nature. 2024 Feb 14;626(7999):529–34. doi: 10.1038/s41586-023-06978-6 (PMC10866709; doi:10.1038/s41586-023-06978-6)
Supplement: Supplementary file 1 — Supplementary Notes 1–3 and Supplementary Figs 1–11. [file 41586_2023_6978_MOESM1_ESM.pdf]

nature portfolio

https://doi.org/10.1038/s41586-023-06978-6

Supplementary information

A 2D ferroelectric vortex pattern in twisted $BaTiO_3$ freestanding layers

In the format provided by the authors and unedited

nature portfolio

https://doi.org/10.1038/s41586-023-06978-6

Supplementary information

A 2D ferroelectric vortex pattern in twisted $BaTiO_3$ freestanding layers

In the format provided by the
authors and unedited

Supplementary Information

A 2D ferroelectric vortex pattern in twisted $BaTiO_3$ freestanding layers

G. Sánchez-Santolino, V. Rouco, S. Puebla, H. Aramberri, V. Zamora, M. Cabero, F. A. Cuellar, C. Munuera, F. Mompean, M. Garcia-Hernandez, A. Castellanos-Gomez, J. Íñiguez , C. Leon, J. Santamaria.

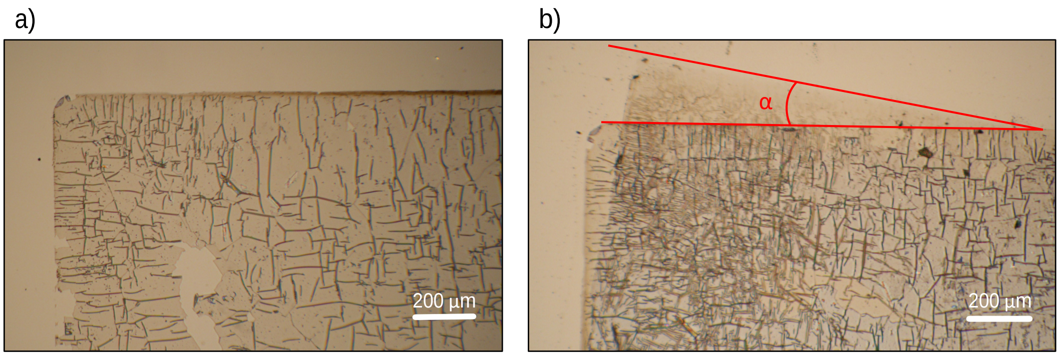

Supplementary Figure S1: Optical microscope images of twisted $BaTiO_3$ freestanding film. a) Optical microscope image of a a) Layer edges are aligned with the [100] substrate direction. b) Twisted $BaTiO_3$ freestanding bilayer with 15 nm thick individual layers. The angle between layer edges (corresponding to [100] crystallographic directions of the individual layers) allows the definition of the twist angle.

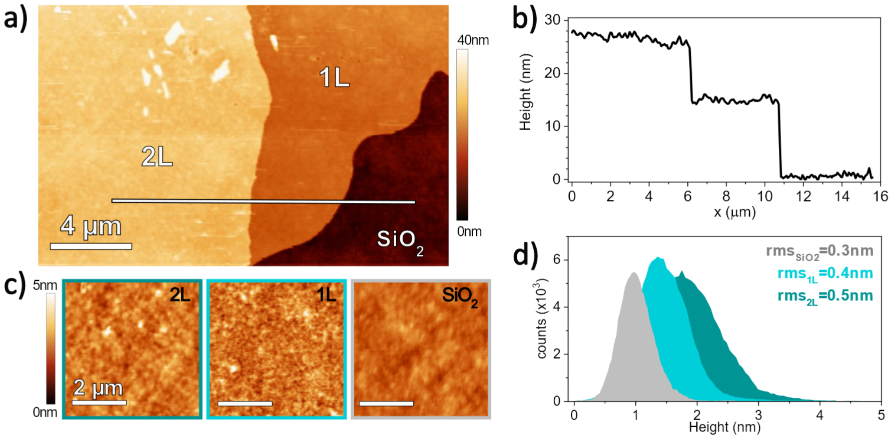

Supplementary Figure S2: AFM images of freestanding $BaTiO_3$ with 7 º twisted layers placed on top of a (TEM) holey Si membrane. a) AFM image of a twisted BTO sample on SiO_2 substrate. Three regions are distinguished: substrate, bottom (1L) and top (2L) BTO layers. b) Line profile obtained from the linescan indicated in panel a). c) Higher-resolution topographic AFM images of the different regions in the sample. Roughness analysis was conducted on these 5x5 μm^2 images. d) Height histogram and root mean square (rms) values derived from the corresponding images in c).

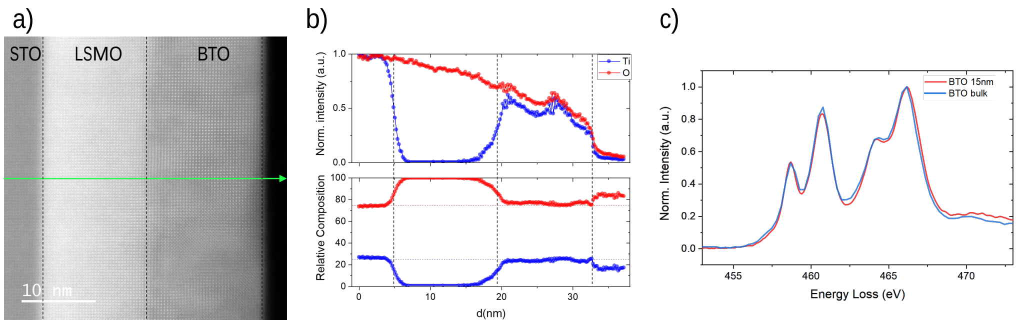

Supplementary Figure S3: STEM-EELS characterization of as grown samples. a) STEM-HAADF image of a $La_{0.7}Sr_{0.3}MnO_3$ / $BaTiO_3$ bilayer grown on top of a $SrTiO_3$ (001) substrate. Green arrow indicates the region where an EELS line scan was acquired. b) EELS intensity profiles showing the effective zero concentration baselines at the LSMO and vacuum regions (top) and elemental composition profiles (in atomic %) for oxygen and titanium obtained from the quantification of the O K and Ti $L_{2,3}$ edges, respectively (bottom). c) Ti $L_{2,3}$ edge of at $BaTiO_3$ thin film compared to the bulk material showing a distinctive Ti +4 edge fine structure. Dashed vertical black lines in (a) and (b) show approximately the position of the interfaces and surface of the bilayer. Overall signal decrease towards the surface indicates a thickness gradient towards the surface which is expected due to sample preparation.

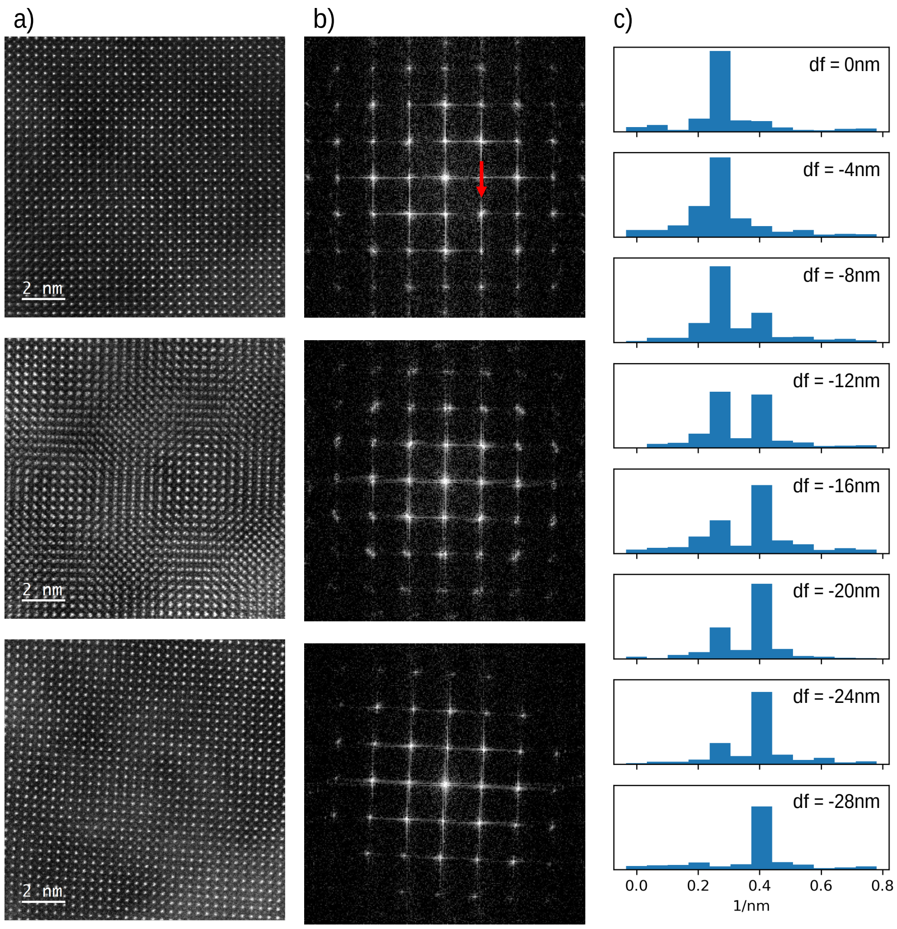

Supplementary Figure S4: Analysis at different focus depths. a) STEM-HAADF images of a 3° twisted $BaTiO_3$ bilayer stack focusing on the top entrance surface (df = 0), the interface of the bilayer (df = -16 nm) and the bottom layer (df = -28 nm). b) Fast Fourier Transform extracted from images in (a). c) Line profiles taken at the [100] reflection (red arrow) at different defocus values of the depth sectioning stack depicting the evolution of the intensity of the spots from both top and bottom layers. For the depth sectioning experiment a series of 20 images were acquired by continuously changing the defocus by a step of 2 nm from the entrance surface of the stack to the exit surface of the stack. Full dataset is shown in Movie S1.

Supplementary Note 1. Toroidal moment

To map the toroidal moment in practice we defined its local value at position r_i, $Q(r_i)$, as $Q(r_i) = \frac{1}{2N}\sum_1^N \Delta r_i x P_i(\Delta r_i)$ where the sum extends to the 24 first and second neighbors of pixel r_i at relative positions Δr_i. This procedure produces lateral averaging and reduces noise at a scale larger that the characteristic size of the topological features (which are consequently not smeared out by the averaging). Toroidal moment alternates sign periodically in diagonal directions of the Moiré pattern (see Figure 2 c and f) in a way determined by a periodic array of alternating clockwise and counterclockwise vortices in AA and AB sites, respectively. Ferroelectric vortices are topological objects characterized by a discontinuous rotation of the polarization around the vortex core in a way that the contour integral of the variance $(\nabla\theta)$ of the orientation on any closed contour *C* enclosing the vortex core divided by 2π (winding number, *n*) is $n = \frac{1}{2\pi}\oint_C \nabla\theta \, dl = +1$. Clockwise and counterclockwise vortices, despite having opposite polarity, do share the same n=+1 winding number. Values of the toroidal moment at vortex sites depend on the size of the vortex and on the ferroelectric displacements (dipole moment). In vertical (and horizontal) directions of the Moiré pattern vortices alternate with antivortices, topological structures with n=-1 winding number and zero toroidal moment. Near the antivortex, polarization curls creating a two-in-two-out configuration of electric polarization vectors.

Supplementary Note 2. Strain and Polarization analysis of the cross-section sample

Since planar views do not supply information about the out-of-plane component of the polarization, the analysis of the cross-section sample supplies complementary information to planar views as it provides a lateral view of the vortex lattice, where the in-plane (P_x along the [1,0,0] direction) and out-of-plane (P_z along the growth direction) components of the polarization vector can be probed, as well as the ε_{xx} in-plane component of the strain tensor. As discussed below, by comparing planar views and cross-section images, we have found a lateral modulation of strain and polarization consistent with the rotational strain

and polarization landscape induced by the Moiré patterns. However, this comparison has to be done taking into account that cross-section images will be averages over the 5 nm depth of focus of the microscope along the [0,1,0] depth of the cross-section sample. Profiles of the planar views have been done averaging the corresponding (Px or ε_{xx}) quantity over the same 5 nm depth of focus. See Supplementary Figure S5 showing a comparison between the lateral modulation of the Px polarization component and ε_{xx} in-plane component of the strain tensor for (5 nm averaged) planar views and for cross-section images (lateral view of the vortex lattice), showing that they both show the same modulation period of 2.3 nm corresponding to the separation between contiguous vortices.

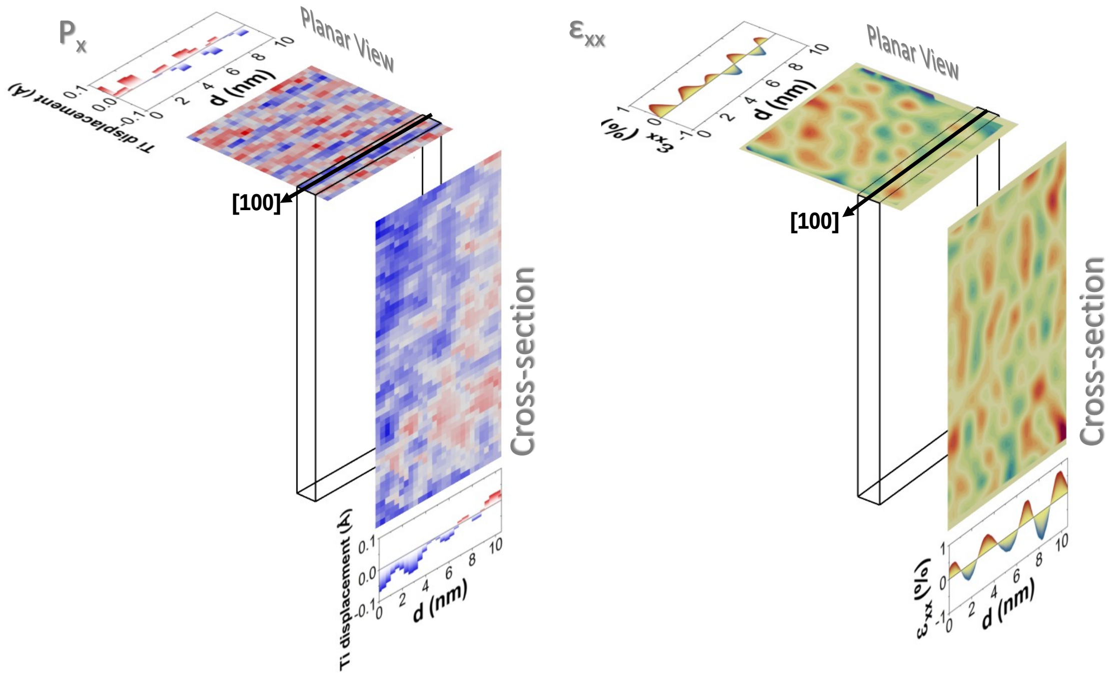

Supplementary Figure S5 Strain and in-plane polarization analysis of cross-section image (top layer) of a 10° twisted $BaTiO_3$ bilayers. In-plane modulation of the Px polarization component obtained from Ti displacements ($d_{Ti} - \langle d_{Ti} \rangle$) (left panel) and in-plane ε_{xx}component of the strain tensor (right panel) for the planar-view and cross-section image of a 10° twisted $BaTiO_3$ bilayer. Profiles show lateral modulation along the [1,0,0] direction (at a 5º angle with the quasi cubic Moiré lattice) of polarization and strain. Notice the close correspondence between the lateral modulations of planar and cross-section views.

Supplementary Figure S6 shows longer scale patterns of ε_{xx} (Panel a) and Px (Panel b) of the cross-section sample of Supplementary Figure S5 together with those of the planar-views (panels c and d), and the corresponding profiles showing a modulation period which coincides roughly with the distance between vortices.

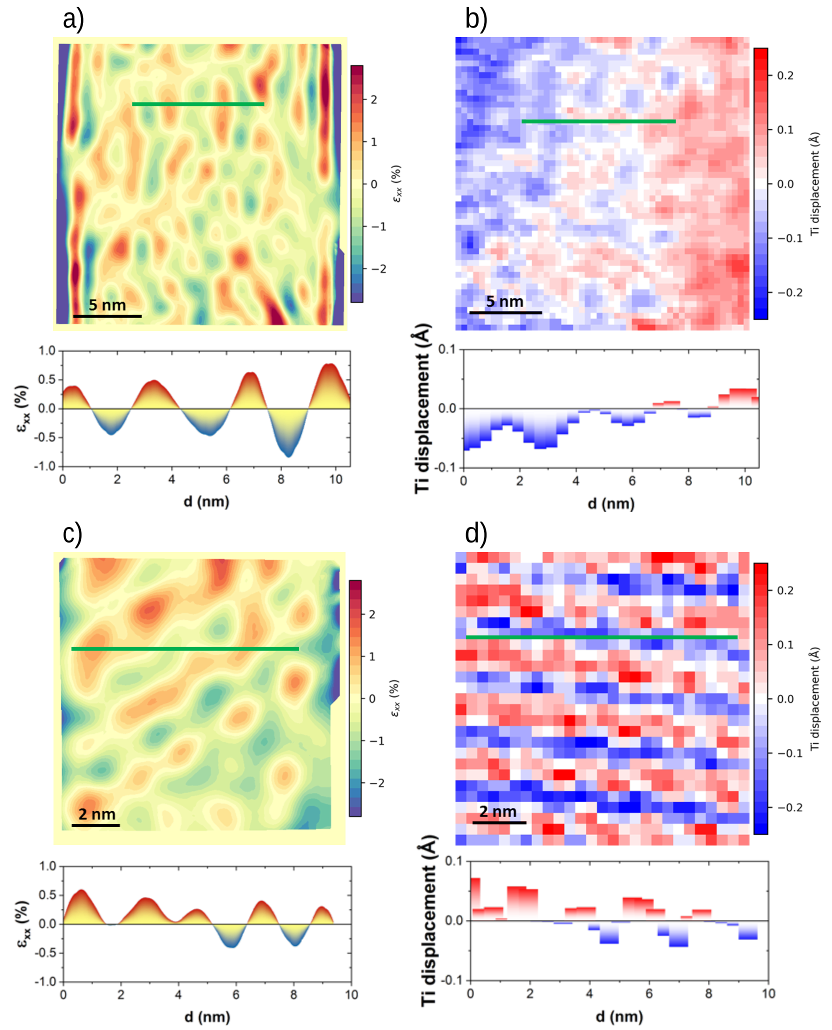

Supplementary Figure S6 In-plane strain and polarization analysis of the top layer of a cross-section sample of 10° twisted $BaTiO_3$ bilayers. a) In-plane ε_{xx} component of the strain tensor and b) in-plane Ti displacement map obtained after subtracting its average value $(d_{Ti} - \langle d_{Ti} \rangle)$ measured at the top layer of a 10° twisted $BaTiO_3$ bilayer cross-section sample. Averaged profiles on the bottom depict the modulations due to the vortex topology. c) In-plane ε_{xx} component of the strain tensor and d) in-plane Ti displacement map obtained after subtracting its average value $(d_{Ti} - \langle d_{Ti} \rangle)$ measured at the planar-view (top layer) image of the 10° twisted $BaTiO_3$ bilayer shown in Fig. 1 of the main text. Averaged profiles on the bottom depict the modulations due to the vortex topology.

We have also found a lateral modulation of the out-of-plane Pz polarization component with a periodicity determined by the vortex lattice. Supplementary Figure S7 shows the lateral modulation of Pz (panels a and b) and Px (panels c and d). Pz polarization maps after subtracting local averages of the polarization over the domains of Extended Data Fig. 6, showed a (short ~2nm) lateral modulation with the same periodicity found for Px roughly corresponding to the distance between vortices. It is tempting to ascribe the out-of-plane polarization to the vortex cores as found in the first principles calculations, however, the finding of a homogeneous polarization state (superimposed to the vortex state) with an out-of-plane component makes this assignment doubtful (as a local increase of the out-of-plane component may simply result from a reduced in-plane polarization to keep the total polarization vector constant). The long period (L_p) modulation results from the 5° misalignment of the [100] crystalline direction of the top layer with the $[100]^M$ direction of the quasi cubic Moiré lattice. Since cross-section images are probing the lateral views of the vortex lattice in the crystalline [100] direction, the long period results from the lateral distance from a vortex along the crystalline [100] direction to find another vortex with the same orientation. I.e., tan 5°=2 nm/ L_p; it follows that the long-period L_p = 22.8 nm.

Yet, it becomes clear that the perspective offered by polarization analysis of the cross-section images is fully consistent with the presence of the vortex lattice found in planar views.

Moreover, cuts at different z-heights consistently showed very similar modulations (both laterally along Px and vertically Pz. See Supplementary Figure S7), confirming that vortices are long (vertically correlated) objects extending along growth direction.

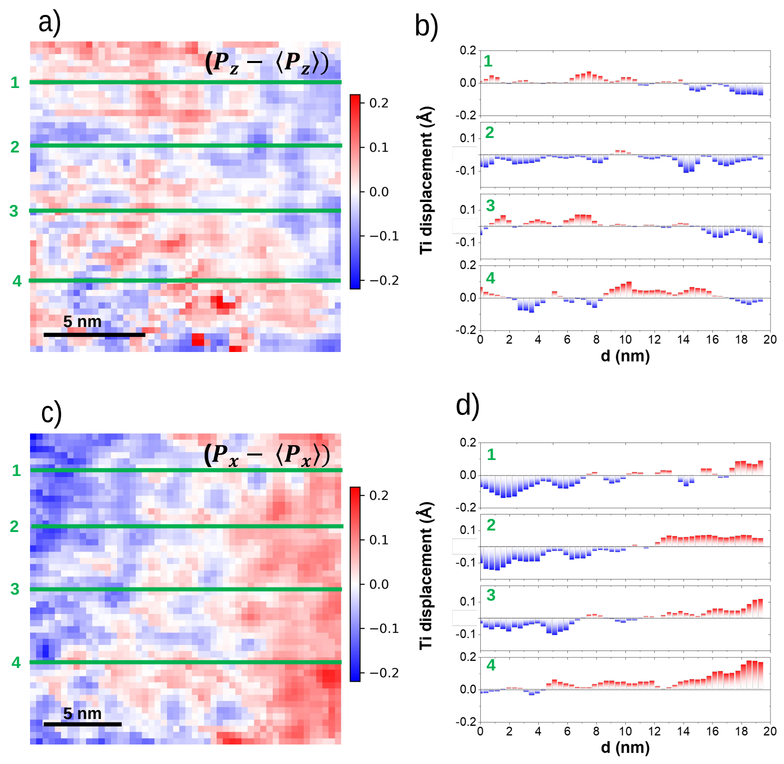

Supplementary Figure S7 Lateral modulation of the Pz and Px polarization components measured at different heights of the top layer of a cross-section sample of 10° twisted $BaTiO_3$ bilayers. a) Out-of-plane Ti displacement $(d_{Ti} - \langle d_{Ti} \rangle)$ measured at the top layer of a 10° twisted $BaTiO_3$ bilayer cross-section sample. b) Out-of-plane Ti displacement $(d_{Ti} - \langle d_{Ti} \rangle)$ profiles obtained along the green lines in (a) at different heights depicting the modulations due to the vortex topology. c) In-plane Ti displacement $(d_{Ti} - \langle d_{Ti} \rangle)$ map measured at the top layer of a 10° twisted $BaTiO_3$ bilayer cross-section sample. d) In-plane Ti displacement $(d_{Ti} - \langle d_{Ti} \rangle)$ profiles obtained along the green lines in (c) at different heights depicting the modulations due to the vortex topology. Notice that data of panel c) coincide with that of panel b) of Supplementary Figure S6.

Supplementary Note 3. Analysis of artefacts in strain and polarization determination.

<u>Image artefacts due to scanning distortions and aberrations.</u>

Occasionally, slightly elliptical atomic columns were observed originating at image artifacts such as scanning distortions or residual aberrations. To account for these possible artifacts in our measurements, we performed systematic image simulations, as shown in Supplementary Figure S8. The STEM-HAADF image simulations were performed using the μSTEM suite[71] with a 200 kV probe, a 30 mrad convergence semi-angle and a HAADF detector with a 70 mrad inner angle and a 200 mrad outer angle, reproducing our experimental conditions. We have included 2-fold (A1) astigmatism and axial coma (B2) aberrations,

for which day-to-day fine tuning is usually performed. For modelling purposes, image simulations were done using a tetragonal $BaTiO_3$ unit cell with polarization aligned along [100].

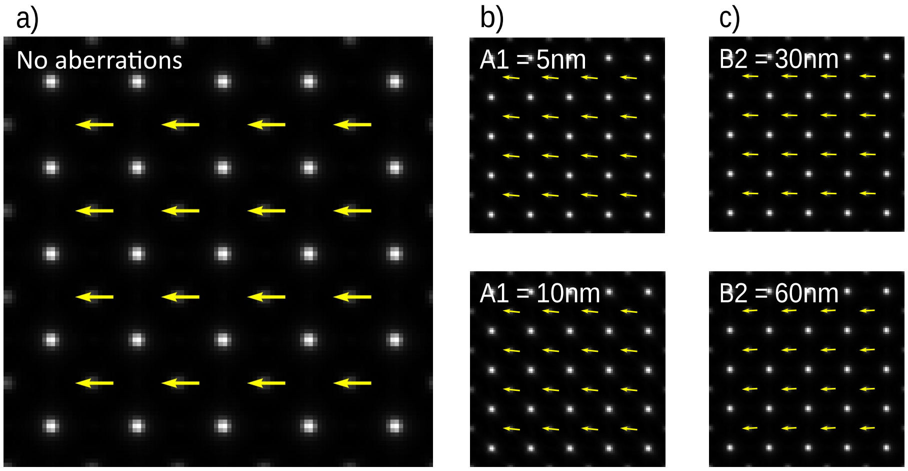

Supplementary Figure S8: Analysis of residual aberrations effects on ferroelectric polarization measurements. a) STEM-HAADF image simulation of a 15 nm tetragonal $BaTiO_3$ layer along the [001] direction for an aberration-free probe focused on the entrance surface (df = 0 nm). b) STEM-HAADF image simulations including a 2-fold astigmatism coefficient of A1 = 5 nm (top) and A1 = 10 nm (bottom) at a 45° azimuth angle. c) STEM-HAADF image simulations including an axial coma coefficient of B2 = 30 nm (top) and B2 = 60 nm (bottom) at a 45° azimuth angle. Ti displacements are amplified by a factor of 20 for clarity.

To simulate artefacts related with aberrations, aberration coefficient with values up to twice the value of the confidence interval, as determined using the CESCOR corrector software, have been included in the simulations. The simulated images show that the measurement of the Ti displacements, and hence, the polarization, is essentially not affected by probe aberrations within the confidence interval. For coefficient values twice as large as the confidence interval there is indeed a small effect on the direction of the polarization. In any case, our simulations (see Supplementary Figure S8) show that the effect of a residual aberrations is homogeneous and cannot produce chiral polarization landscapes, as the ones shown on this manuscript.

FFT methods for determination of strain. Strain patterns were checked for systematic errors reported for FFT methods like the Geometric Phase Analysis (GPA) when applied to materials with multi atom unit cells. We have performed a strain analysis of a 11° twisted 30 nm BTO bilayer using different methods including the Peak Pair Analysis (PPA) used throughout the manuscript, the Geometric Phase Analysis (GPA)[72] and an estimation of the strain from our atomic position mapping analysis.

As described in Peters et. al. [72], in GPA analysis of strain, contrast modulation in atomic resolution STEM-HAADF images with two atoms (or more) per cell can result in non-zero "apparent" strain values even in absence of true strain. According to Peters et. al. [72], in our case with images with two visible sublattices (A:Sr and B:Ti sites), such artefacts can be avoided by choosing g-vectors for the GPA analysis that meet the relation $g \cdot v = n$, where $v = \frac{1}{2}[110]$ is the displacement vector between A and B sublattices and n is an integer. In Extended Figure S9 we show the strain analysis results for the ε_{xx} and ε_{xy} components of the lattice strain tensor using the vectors $\boldsymbol{g} = (100)$ and $\boldsymbol{g} = (010)$ used in the strain analyses presented in our manuscript (panels and vectors $\boldsymbol{g} = (110)$ and $\boldsymbol{g} = (1\bar{1}0)$ which meet the afore mentioned relation, along with the results of both the GPA and PPA analysis using the vectors. The comparison of these strain maps shows very similar strain modulations, all following the periodicity of the Moiré corresponding to an 11° twisted bilayer.

To rule out any effects on our strain analysis from other types of image contrast modulations (driven by the FFT method) due to the Moiré structure, we have directly estimated the strain along the xx direction from the atomic position mapping measurements used for the polarization analysis in this same image. We have estimated the strain from the relative change of the lattice parameter (computed from the images at each unit cell) from its mean value over whole image. This analysis is shown in Supplementary Figure S9 h) l), p) and t) and depicts the same lattice modulation as the GPA and PPA strain analysis regardless of the chosen g-vectors.

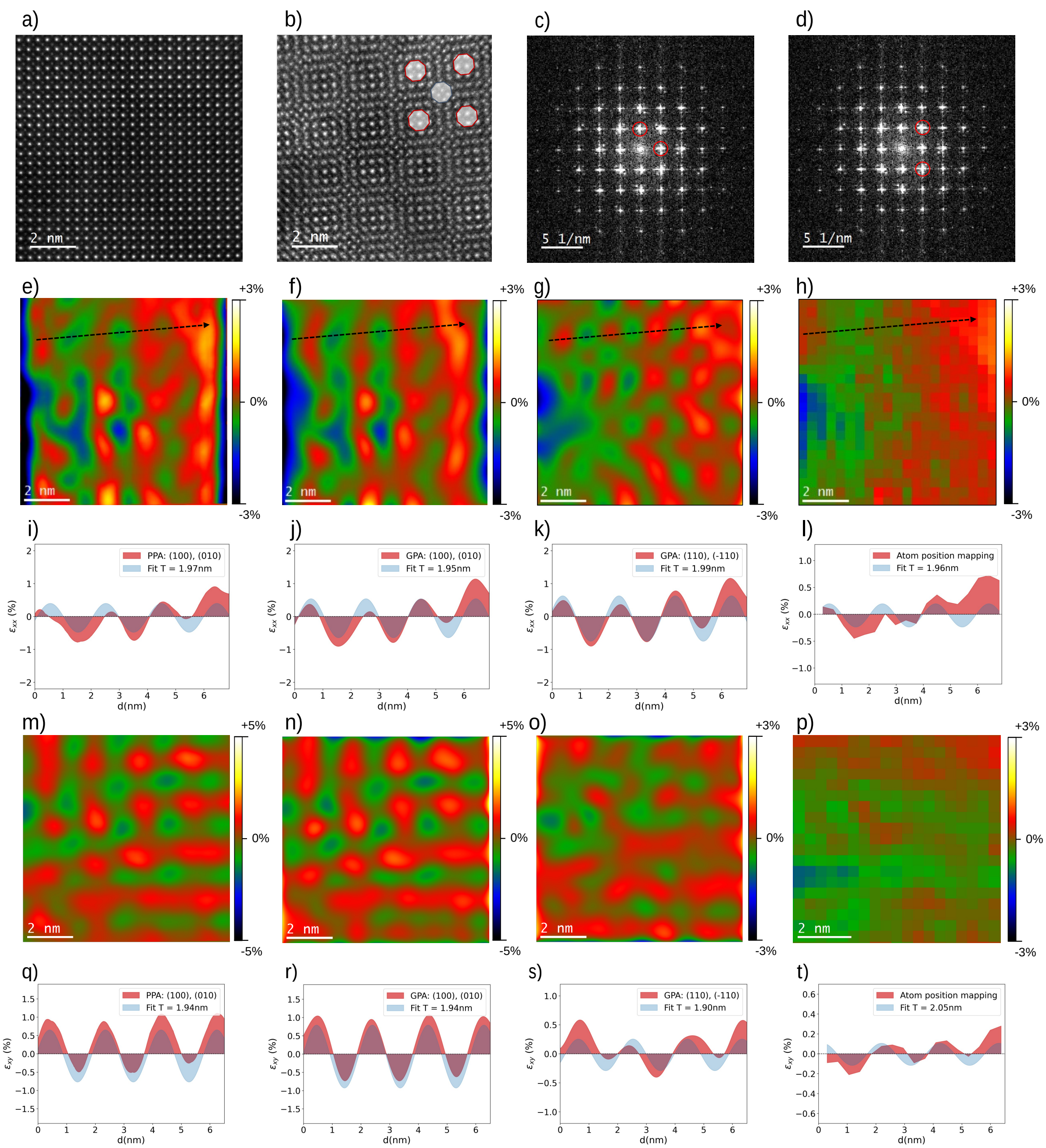

Supplementary Figure S9: Strain analysis comparison for a twisted 30nm $BaTiO_3$ bilayer. a) STEM-HAADF (planar view) image of a 11° twisted $BaTiO_3$ bilayer stack focusing on the top entrance surface (defocus = 0 nm). b) STEM-HAADF (planar view) image focusing on the interface of the bilayer (defocus = -30 nm). c) FFT of the image in (a) marking the (100) and (010) spots used for GPA and PPA analysis. d) FFT of the image in (a) marking the (110) and (-110) spots used for GPA analysis. e) ε_{xx} component of the lattice strain tensor obtained using the (110) and (-110) spots for GPA. f) ε_{xx} component of the lattice strain tensor obtained using the (100) and (010) spots for GPA. g) ε_{xx} component of the lattice strain tensor obtained using the (100) and (010) spots for PPA. h) ε_{xx} lattice strain calculated from the atomic position mapping using Atomap. i), j), k), l) show the profiles along the black arrow in panels e) to h). m) ε_{xy} component of the lattice strain tensor obtained using the (110) and (-110) spots for GPA. n) ε_{xy} component of the lattice strain tensor obtained using the (100) and (010) spots for GPA. o) ε_{xy} component of the lattice strain tensor obtained using the (100) and (010) spots for PPA. p) ε_{xy} lattice strain calculated from the atomic position mapping using Atomap. q), r), s), t) show the profiles along the black arrow in panels m) to p).

An additional evidence that strain patterns in our twisted layers do not result from analysis artefacts can be obtained from the comparison between the strain results of planar-view images of freestanding twisted BTO bilayers and the ones obtained from the analysis of cross-sectional bilayers, as shown in Supplementary Figures S5 and S6, in which the same modulation of the in-plane strain component is found.

This analysis of strain in our STEM-HAADF images demonstrates that contrast-forming mechanisms cannot be responsible for the lattice modulations found in our twisted BTO bilayers and indeed, our measurements describe real physical effects.

Channeling effects. To study the possible influence of channeling effects on the measurements of polarization displacements in twisted $BaTiO_3$ bilayers, we have conducted multislice electron scattering simulations.

The STEM-HAADF image simulations were performed using the μSTEM suite [71] with a 200 kV probe, a 30 mrad convergence semi-angle and a HAADF detector with a 70 mrad inner angle and a 200 mrad outer angle, reproducing our experimental conditions. Two image simulations were carried out using two tetragonal $BaTiO_3$ 10x10x37 (4x4x15 nm) supercells with the polarization aligned along the [100] direction and twisted by 3° and 10° degrees respectively. For comparison with our experimental results, the defocus value was set at top layer entrance surface (df = 0 nm) and the same procedure as with the experimental images was used to map the polarization displacements. Supplementary Figure S10 compares the simulated effects of channeling with the experimentally observed vortex array. Profound differences can be readily appreciated.

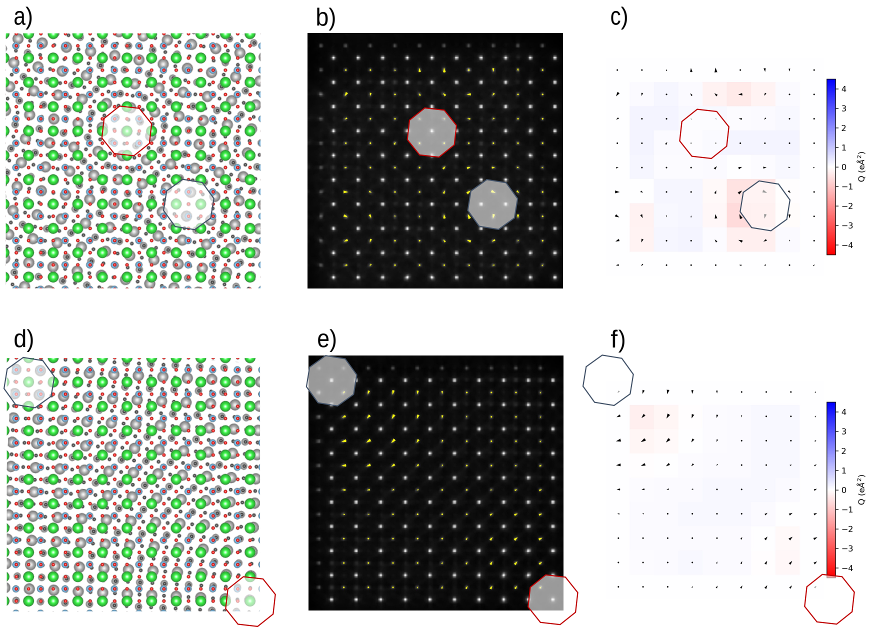

Supplementary Figure S10 Simulated STEM-HAADF image analysis of 3° and 10° twisted $BaTiO_3$ bilayers. a) Model of the rigid atomic structure corresponding to two $BaTiO_3$ lattices with a twist angle of 10° showing Ba atoms in green, Ti in blue and O in red for the top layer and in grey for the bottom layer b) STEM-HAADF simulated image of a 10° twisted $BaTiO_3$ bilayer with the defocus set at the top layer entrance surface (df= 0nm). c) Ti displacement map $(d_{Ti} - \langle d_{Ti} \rangle)$ (black arrows) measured on the simulated STEM-HAADF image after subtracting the homogeneous component, superimposed to the contour plot of the toroidal moment (Q) of the ferroelectric polarization. Ti displacements are amplified by a factor of 20 for clarity. d), e), f) show the same analysis for a STEM-HAADF simulated image of a 3° twisted $BaTiO_3$ bilayer. AA and AB sites are marked in red and blue, respectively.

Image simulations show that channeling effects modify the homogeneous polarization landscape only very weakly. Although a very small rotational polarization component may be observed locally after subtracting the homogeneous polarization, its magnitude is at best 0.03 Å compared to the 0.12 - 0.15 Å found experimentally at vortex sites. I.e., as opposed to the experimental vortex array where the full ferroelectric moment is observed to wind around vortex sites, channeling effects would account only for a small fraction of it. As a result, local values of the toroidal moment obtained in the simulations are correspondingly very small: they are at most 0.25 eÅ^2 compared to 4 eÅ^2 found experimentally at vortex sites. Finally, the simulated polarization pattern resulting from channeling effects is geometrically very different from the vortex array observed experimentally, not showing the highly correlated arrays of vortices (with both helicities) and antivortices. We conclude that channeling effects pose a 6 % error bar to the observed vorticity as described by the toroidal moment.

Further support to the negligible effect of channeling effects can be obtained from the analysis of fast Fourier transformed images obtained from the depth sectioning experiment shown in Supplementary Movie S1 while continuously changing defocus from the top to the bottom surface. See Supplementary Figure S4. Intensity line profile at the (100) spot show that on the images focusing on the top surface (df = 0 nm) or on the bottom surface (df = -28 nm), there is no intensity from the (100) spot of the other layer. For intermediate defocus values intensity corresponding to both layers can be observed. In particular, for a focus depth of df = -14 nm clearly resolved contributions of both layers with comparable intensity (see Extended Data Fig. 4) allow for an accurate determination of the twist angle of the bilayer. Notice, however, that for a defocus value of df = -8 nm, we start seeing signal corresponding to the bottom layer. This analysis agrees with previous estimates based on multislice simulations of a depth of field of 55 Å at the best for our experimental conditions (200kV acceleration voltage and 30 mrad probe forming aperture) [49]. This allowed taking images from the top and from the bottom surface free of artifacts due to the mixing of intensity contributions from both layers (provided layer thickness is larger than the depth of field), but it precludes a systematic depth resolved analysis. Strain and polarization analysis could be conducted at both (top and bottom) surfaces, and both showed unambiguously similar strain patterns and a chiral polarization landscape featuring vortices and antivortices. See Supplementary Figure S11. Notice the close similarity between ferroelectric topologies of top and bottom layer. Unfortunately, the spatial correspondence cannot be assessed since changes in focus and image conditions do not allow to quantify a possible drift between images.

The observation of similar vortex topologies at top and bottom surfaces of the bilayer further excludes artifacts due to channeling of the electron beam which is expected to be maximal at the images of the top surface and nearly absent at the images of the bottom surface.

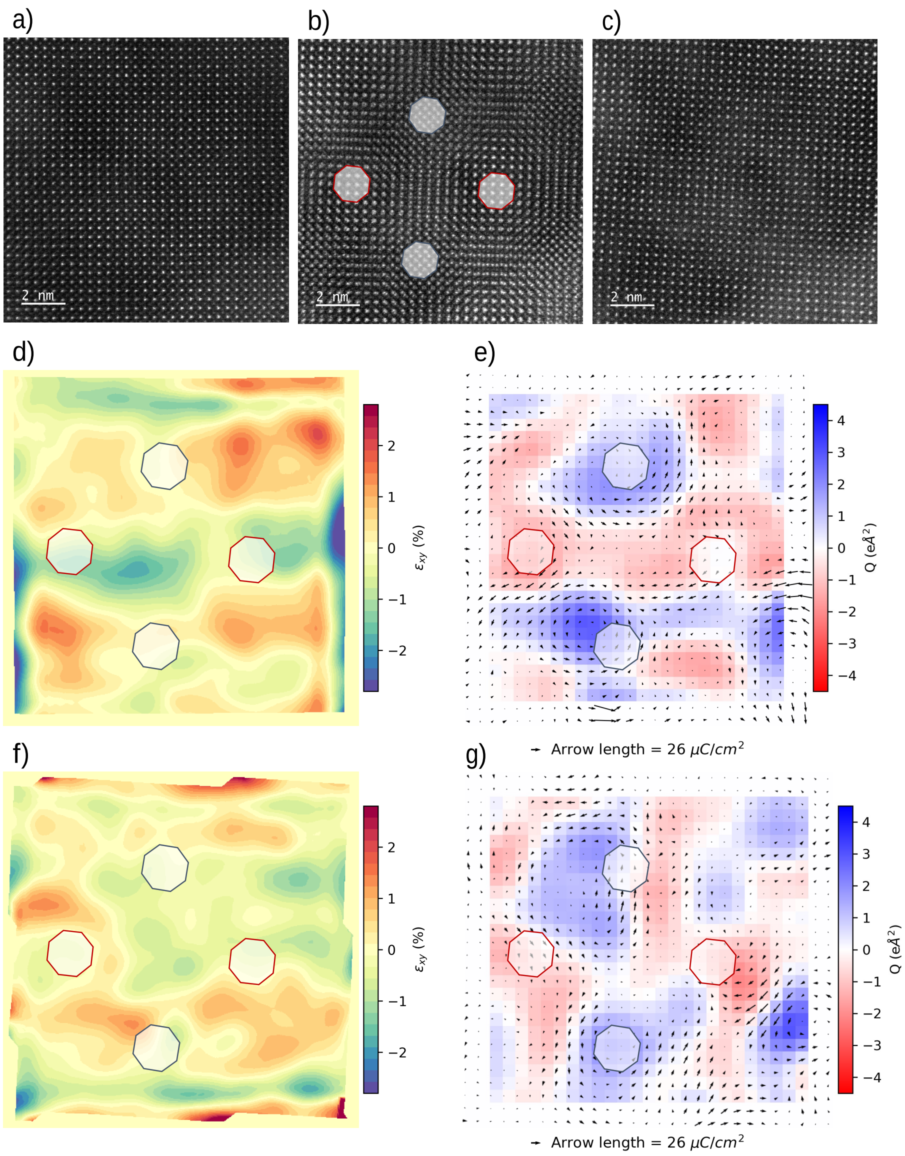

Supplementary Figure S11: Strain and polarization modulations at top and bottom surfaces of twisted $BaTiO_3$ bilayers. STEM-HAADF (planar view) images of a 3° twisted $BaTiO_3$ bilayer stack focusing on a) the top entrance surface (df = 0), b) the interface of the bilayer (df = -15 nm) and c) the bottom layer (df = -28 nm). Shear strain (ε_{xy} component of the lattice strain tensor) at d) the top $BaTiO_3$ layer and f) the bottom $BaTiO_3$ layer. Ti displacement map ($d_{Ti} - \langle d_{Ti} \rangle$) after subtracting a homogeneous component (black arrows) measured on e) the top $BaTiO_3$ layer and g) the bottom $BaTiO_3$ layer, corresponding to the same area superimposed to the toroidal moment (Q) of the ferroelectric polarization. Ti displacements are amplified by a factor of 20 for clarity. Red and blue marks in all panels indicate the AA and AB stacking regions, respectively. There is a slight misalignment between the top and bottom layer images due to sample drift.

Supplementary Movie S1: Depth sectioning experiment. For the depth sectioning experiment, a series of 20 STEM-HAADF images of a 3° twisted 15 nm $BaTiO_3$ bilayer were acquired by continuously changing the defocus by a step of 2 nm from the entrance surface of the stack to the exit surface of the stack.
